# Structural basis for heterogeneous phenotype of ERG11 dependent Azole resistance in *C.albicans* clinical isolates

**DOI:** 10.1186/2193-1801-3-660

**Published:** 2014-11-06

**Authors:** Surajit Debnath, Soma Addya

**Affiliations:** Department of Medical Laboratory Technology, Women’s Polytechnic, Hapania, Tripura (W) India; Government of West Bengal, Paschim Medinipur, West Bengal, India

**Keywords:** *Candida albicans*, Drug resistance, ERG11, Mis-sense mutations, Active site geometry, Vital catalytic residues, Protein energetics, Protein dynamics

## Abstract

Correlating antifungal Azole drug resistance and mis-sense mutations of ERG11 has been paradoxical in pathogenic yeast *Candida albicans.* Amino acid substitutions (single or multiple) are frequent on ERG11, a membrane bound enzyme of Ergosterol biosynthesis pathway. Presence or absence of mutations can not sufficiently predict susceptibility. To analyze role of mis-sense mutations on Azole resistance energetically optimized, structurally validated homology model of wild *C.albicans* ERG11 using eukaryotic template was generated. A Composite Search Approach is proposed to identify vital residues for interaction at 3D active site. Structural analysis of catalytic groove, dynamics of substrate access channels and proximity of Heme prosthetic group characterized ERG11 active site. Several mis-sense mutations of ERG11 reported in *C.albicans* clinical isolates were selected through a stringent criterion and modeled. ERG11 mutants subsequently subjected to a four tier comparative biophysical analysis. This study indicates (i) critical interactions occur with residues at anterior part of 3D catalytic groove and substitution of these vital residues alters local geometry causing considerable change in catalytic pocket dimension. (ii) Substitutions of vital residues lead to confirmed resistance in clinical isolates that may be resultant to changed geometry of catalytic pocket. (iii)These substitutions also impart significant energetic changes on *C.albicans* ERG11 and (iv) include detectable dynamic fluctuations on the mutants. (v)Mis-sense mutations on the vital residues of the active site and at the vicinity of Heme prosthetic group are less frequent compared to rest of the enzyme. This large scale mutational study can aid to characterize the mutants in clinical isolates.

**Electronic supplementary material:**

The online version of this article (doi:10.1186/2193-1801-3-660) contains supplementary material, which is available to authorized users.

## Background

*Candida albicans* is an opportunistic fungal pathogen that causes various mucosal infections (Ge et al. 2010) in general population and life-threatening systemic infections in immuno compromised patients (Feigal et al. 1991, Richardson & Lass-Florl 2008). The pathogenic yeast has been exposed to its conventional therapy of Azole drugs for a considerable period of time due to longer dosage regime in patients with deranged immunity. This along with it’s over the counter use for topical applications have lead to Azole resistance, in *C.albicans*, a strategy to increase fitness against a constant challenge, a simple evolutionary phenomenon. Azole drugs target 14a-Lanosterol Demethylase (ERG11), a unique heme-thiolate enzyme (Pfam: P450, amino acid range 49–520) of the fungus and competitively inhibits it. ERG11 catalyses two successive hydroxylations of the 14alpha-methyl group, followed by its elimination as a Formate moiety, leaving a 14(15) double bond (KEGG**) -** a key step in biosynthesis of Ergosterol (Klein et al. 1984). As a vital membrane lipid Ergosterol provides rigidity, stability, and stress resistance (Prasad and Kapoor 2004) to the fungal cell and loss of which leads to cell lysis. Though similar to cholesterol synthesis in mammals the pathway for Ergosterol biosynthesis differs in some obvious ways (Hitchcock, 1993) and so ERG11 was targeted for management of this eukaryotic pathogen in its eukaryotic human host.

Because of the safety profile (Schweitzer et al. 1996) and high therapeutic index, Azoles have been the drug of choice for many years as first-line therapy, antifungal prophylaxis and empirical or preemptive treatment (Morio et al. 2010). Two classes of Azoles are in use (1) the Imidazoles in topical infections and (2) Triazoles in systemic infections (Sanglard et al. 2009). The fungal pathogen has developed resistance against these antibiotics by employing a variety of molecular strategy (Franz et al. 1998, Sanglard et al. 1998a, 1998b, Sanglard et al. 2003, White 1997a, 1997b, White et al. 2002). Mutations in the ERG11 gene (leading to reduced affinity of the enzyme to Azoles) is the most perceived mechanism of resistance (Kelly et al. 1999 [a], [b], Löffler et al. 1997, Sanglard et al. 1998a, 1998b, White 1997a, 1997b). However several issues on the role of ERG11 in Azole resistance has raised: (i) More than 140 different amino acid substitutions have been reported in Erg11 of *C. albicans* clinical isolates (Morio et al. 2010), including multiple substitutions occurring simultaneously in various combinations (Favre et al. 1999; Goldman et al. 2004). (ii) The genetic polymorphism suggests that Lanosterol Demethylase is highly permissive to structural changes. (iii) Evidences indicate that amino acid changes in ERG11 do not contribute equally to Azole resistance (Morio et al. 2010). (iv) Several mutations are found in both Azole resistant and susceptible strains (Chau et al. 2004; Kakeya et al. 2000; Lamb et al. 2000; Loffler et al. 1997; Sanglard et al. 1998a, 1998b) so the presence or absence of mis-sense mutation is not sufficient to predict Azole susceptibility (Morio et al. 2010). (v) Single point mutation may or may not drastically affect Azole sensitivity of ERG11 and combinations of point mutations may have cooperative effects (Sanglard et al. 1998a, 1998b). These peculiarities of ERG11 mutations in terms of their varied effect make Azole resistance in *C.albicans* a difficult problem to address.

Among several examples of discrepancy the single substituent D116E has been described in Azole-susceptible as well as Azole-resistant isolates (Chau et al. 2004; Favre et al. 1999; Marichal et al. 1999; Perea et al. 2001; Sanglard et al. 1998a, 1998b; White et al. 2002; Xu et al. 2008). D116E has also been described in combinations in clinical isolates with quadruplet mutation ERG11_D116E_K128T_Y132H_G465S. The mutant has been described in five reduced susceptibility isolates, but the correlation of this pattern with resistance is still uncertain (Ying et al. 2013). Individually occurring A114S (Jiang et al. 2006; Xu et al. 2008) and Y257H (Chau et al. 2004; Xiao et al. 2004; Xu et al. 2008) single point mutations has been isolated in different FLZ resistant starins. These missense mutations also have been reported in combinations, such as ERG11_A114S_Y257H which was identified in resistant as well as susceptible dose-dependent isolates (Ying et al. 2013). Similarly Y132H has been isolated in resistant strains (Chau et al. 2004; Favre et al. 1999; Kakeya et al. 2000; Marichal et al. 1999; Sanglard et al. 1998a, 1998b; Xu et al. 2008) and a cumulative increase in resistance is reported when it occur with other mutations. G450E is reported in resistant strains either singly or in various combinations (Chau et al. 2004; Favre et al. 1999; Goldman et al. 2004; Loffler et al. 1997, Perea et al. 2001). Combined occurrence of two substitutions as ERG11_ Y132H _ G450E is reported in resistant strains (Ying et al. 2013). Some single mutants as K342R that have been isolated only in Azole susceptible strains (Ying et al. 2013;Goldman et al. 2004). In contrast some single mutations are found exclusively in resistant strains, such as P230L (Li et al. 2004; Xiao et al. 2004) and F380S (Goldman et al. 2004). There are some substitutions that are experimentally found to be associated with Azole resistance in *C.albicans* but have not been recovered in clinical isolates, such as T315A, Y118A, Y118F, and Y118T (Baldwin and Kelly 1997; Chen et al. 2007; Lamb et al. 1997).

In this scenario a large scale analysis of various categories of ERG11 mutants is required to gain further insight on the role of the enzyme and its amino acid substitutions in Azole resistance. Over the years we have acquired substantial knowledge on amino acid substitutions on ERG11 but the precise way how the amino acid exchanges influence the drug resistance is not well explained. With more structural information on the wild-type and mutated enzymes the intricacy of ERG11 catalysis and effects of mutations will be fully understood. Since ERG11 is a membrane protein which tends to be resolved poorly by experimental procedure (X-ray,NMR), molecular modeling may be a reliable method for structural studies (Shuang et al. 2007). Earlier studies have attempted ERG11 modeling from prokaryotic templates. Those were either focused on fundamental properties (Lewis et al. 1999) of ERG11 or screening of Azoles (Ji et al. 2000, Shuang et al. 2007) and new pharmacophores (Sheng et al. 2006). In the present work a large-scale structural study of the ERG11 mutants is undertaken in order to comprehend the contradiction in terms of occurrence of mutations with or without Azole resistance in *C.albicans*.

## Results

### The wild type ERG 11 of *Candida albicans*

The wild type ERG 11 of *Candida albicans* was built using 4K0F.PDB (Lanosterol 14-alpha demethylase of *Saccharomyces cerevisiae* strain YJM789 with intact transmembrane domain bound to Itraconazole) resolved at 2.19 Å. Template was selected by Swiss models customized scoring scheme. Alignment of the target (ERG11 of *C.albicans* NCBI Ref Seq: XP_716761.1 of *C.albicans*, strain SC5314) and template 4K0F (ERG11 of *Saccharomyces cerevisiae* strain YJM789) have 66.21% sequence identity. A pairwise alignment between the primary structures (in FASTA) by the Needle program of EMBL revealed a Needle Score of 1815.5. 3D structural difference between a model and its template is conventionally estimated from RMSD value which was 0.20 Å among the final ERG11 model and the template, as estimated by “Iterative Magic Fit” on alpha carbons of the 3D coordinates. There seems to be considerable homology among the target and the template. The wildtype ERG11 protein have a predicted molecular weight of 60675.4 Da and predicted pI is 6.69. The composition of the wildtype ERG11 protein is tabulated (Table 1).

**Table 1:** **Amino acid composition of wildtype ERG11** ***Candida albicans***

Amino acids	Number of residues	Percentage
Ala (A)	25	4.7%
Arg (R)	25	4.7%
Asn (N)	19	3.6%
Asp (D)	32	6.1%
Cys (C)	4	0.8%
Gln (Q)	17	3.2%
Glu (E)	30	5.7%
Gly (G)	32	6.1%
His (H)	13	2.5%
Ile (I)	32	6.1%
Leu (L)	47	8.9%
Lys (K)	35	6.6%
Met (M)	13	2.5%
Phe (F)	33	6.2%
Pro (P)	29	5.5%
Ser (S)	37	7.0%
Thr (T)	32	6.1%
Trp (W)	8	1.5%
Tyr (Y)	29	5.5%
Val (V)	36	6.8%

**Figure 1:**
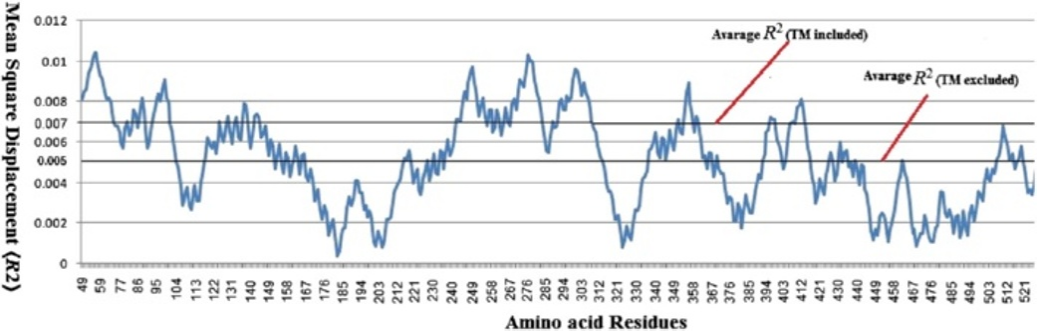
**Mean Square Displacement 〈** ***R*** ^**2**^ **〉 of each residue in the Wild type ERG 11 of** ***Candida albicans.***

Structural quality improvement regime for *C.albicans* wild type ERG11 given most suitable parameters after 40 cycles of steepest descent and the final model quality approached the benchmark values of the template. A detail of the quality improvement regime is given in the Additional file 1. Total energy of the final model was -24909.789 K.J/mole (Table 2) which indicates it to be stable. The catalytic cavity of the final ERG11 can be accessed by two channels and a Heme prosthetic group is in close proximity of the catalytic site. The average Mean Square Displacement 〈*R*^2^〉 of each residue in the Wild type ERG 11 of *Candida albicans* is 0.0071 (including highly flexible Trans-membrane segment) and 0.0051 (Figure 1) (excluding TM). In the Wild type ERG11 model 305 (57%) out of 536 residues remains buried in the protein lattice (NetsurfP).

**Table 2:** **Energetic assessment of the initial and final ERG11** ***Candida albicans*** **model**

Proteins	Energy parameters						
	Bonds energy (KJ/mole)	Angels energy (KJ/mole)	Torsion energy (KJ/mole)	Improper energy (KJ/mole)	Nonbonded energy (KJ/mole)	Electrostatic energy (KJ/mole)	Toal energy (KJ/mole)
Intitial Model Wild ERG11	633.755	3071.161	2037.510	523.60	-12946.40	-13659.73	-20340.652
Final Model Wild ERG11	439.711	3200.458	2128.688	657.486	-16825.04	-14511.09	-24909.789
**Template 4k0f.pdb**	1105.138	2512.448	2736.639	467.556	-14620.22	-13716.33	-21514.770

### Vital amino acids at the active site of the Wild type ERG 11 of *Candida albicans*

**Figure 2:**
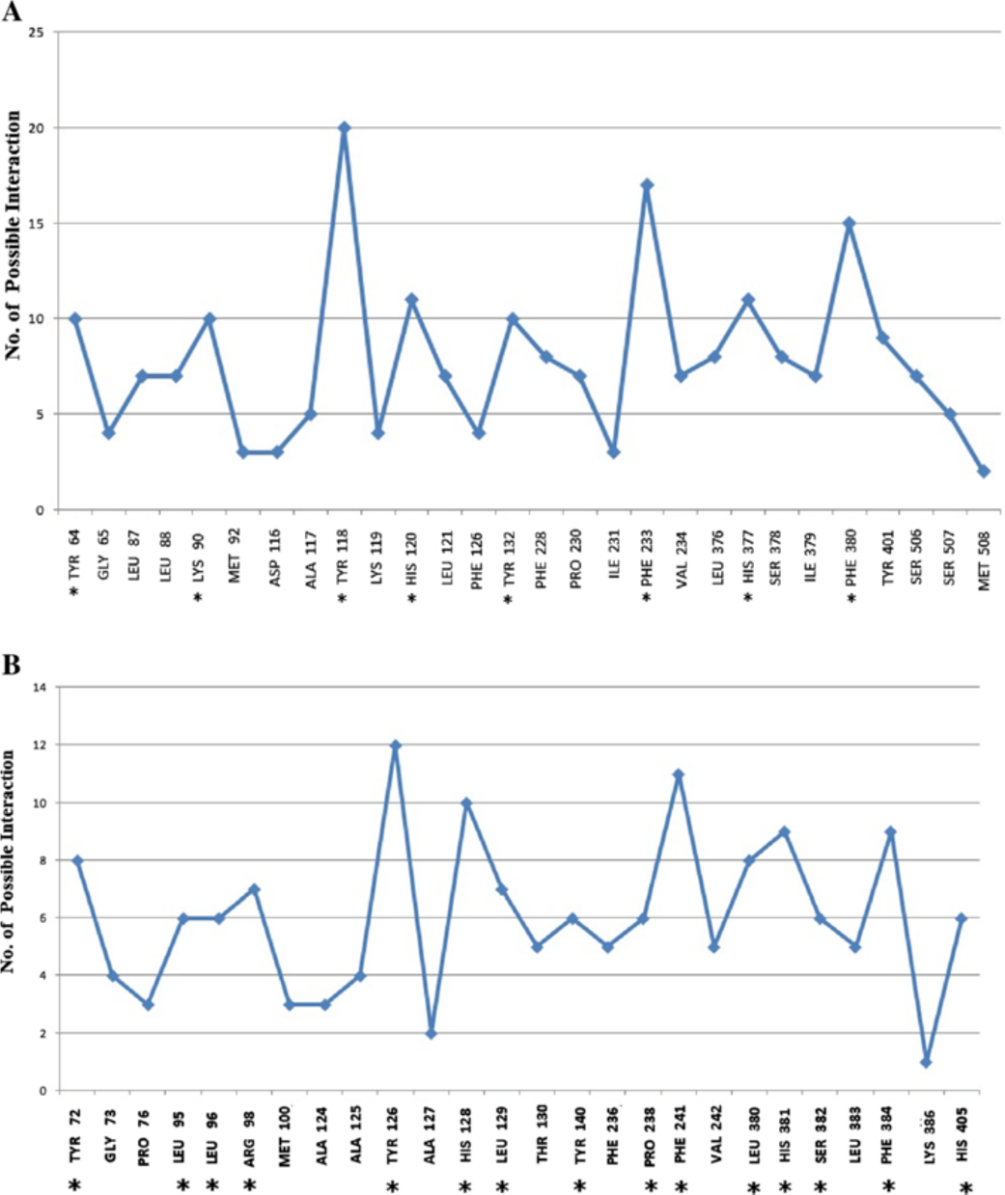
**Active site residues of the Wild type ERG 11 and template 4K0F.pdb showing the number of possible interactions (Q-SiteFinder). A** Active site residues of the Wild type ERG 11 (Amino acid with highest number of interactions (TYR118) and those with a minimum 50% of the highest interactions are star marked). **B** Active site residues of the template 4K0F showing the number of possible interactions (Amino acid with highest number of interactions (TYR126) and those with a minimum 50% of the highest interactions are star marked).

**Figure 3:**
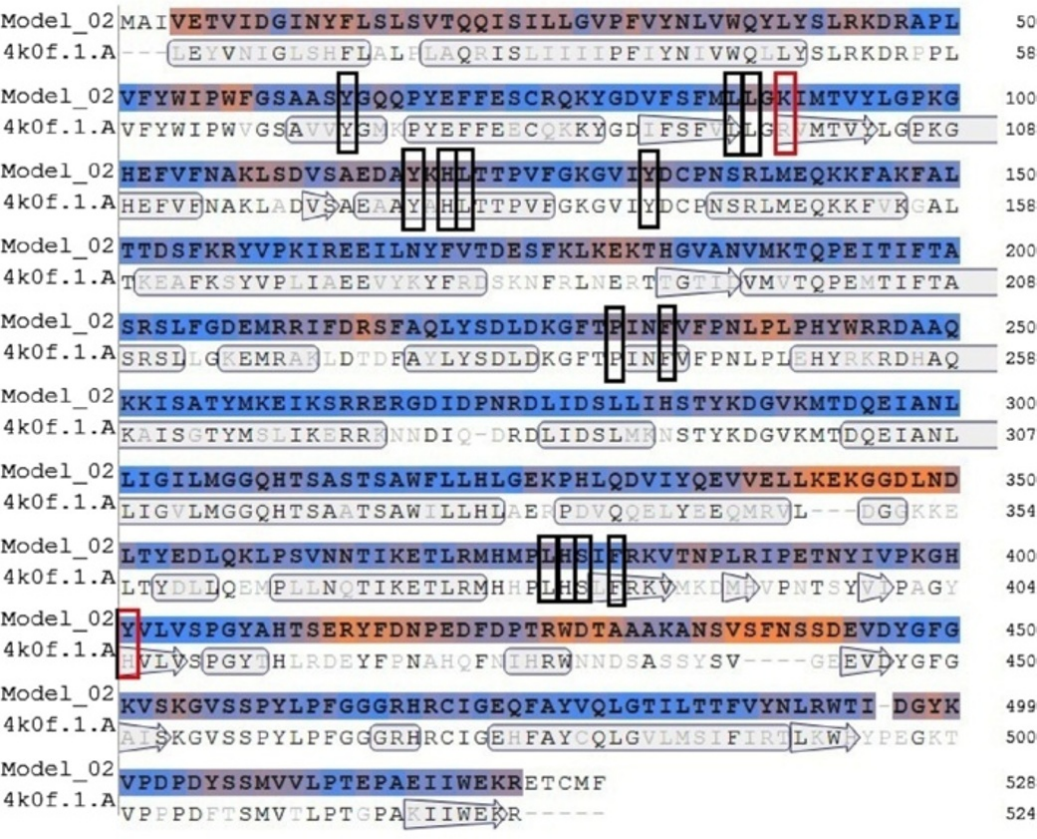
**Alignment of Target (ERG11 of** ***C.albicans*** **) and the template 4K0F showing composite residues that are predicted to be vital for interactions.** SwissModel Sequence Identity 66.21% , Needle Score 1815.5.

**Figure 4:**
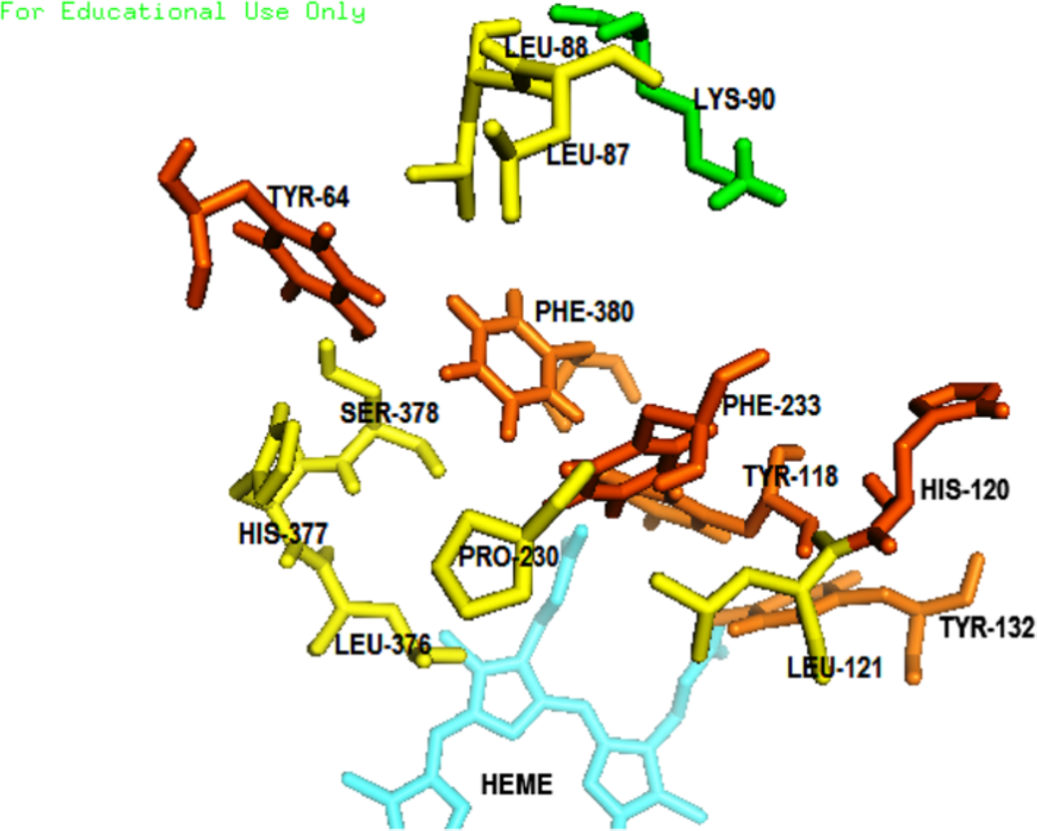
**Composite residues that are predicted to be vital for various interactions in the active site of** ***C.albicans*** **ERG11.**

**Figure 5:**
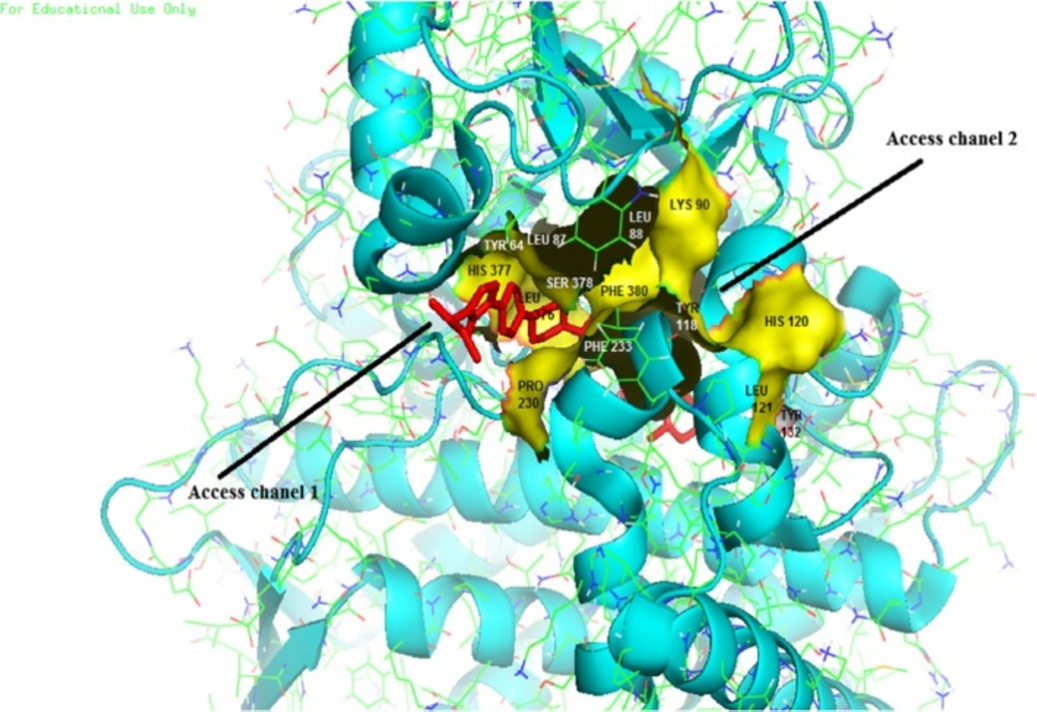
**Superimposed model of ERG11 of** ***C.albicans*** **over the template 4K0F showing the surface of the 14 composite residues.** The two substrate access channels and the Inhibitor (bound to the template) are seen. Template shown in Helix-sheet-loop and the model of ERG11 of *C.albicans* is shown in stick formation.

28 amino acids are predicted to be present on the Wild type ERG 11 active site while 26 amino acids are predicted on the template 4K0F by Q-site finder. Highest number of interactions on any residue are 20 and 12 for the Wild type ERG 11 and the template respectively (Figures 2A, 2B).As per the composite approach assumption 8 residues on the Wild type ERG 11 model and 15 residues on the template are found to be vital for catalysis (star marked residues, Figures 2A, 2B). Vital residues identified on Template (Figure 2B) were traced on the Wild type ERG 11 from the alignment (in FASTA, Figure 3) between Wild type ERG 11 and 4K0F. The residues R98 and H405 of the template correspond to K90 and Y401 respectively of the wild ERG of *C.albicans*. There are 13 composite residues on Wild type ERG 11 of *Candida albicans* as seen in the alignment which are vital for catalysis (Figure 3). K90 was included as vital residue as it makes 10 interactions in the wild ERG11. Therefore it is predicted that at least 14 residues are imperative for interactions at the catalytic pocket of ERG11 of *Candida albicans* (Figure 4). The surface generated upon these 14 vital residues on the superimposed figure shows the accommodating surface of the substrate/inhibitor at the active site (Figure 5) of wild ERG 11 in *Candida albicans*. Vital residues occur at anterior part of the catalytic furrow. Among the predicted 14 vital residues on ERG 11 of *Candida albicans* only two vital residues P230L and F380S has been found to be substituted in Azole resistant single mutant *Candida albicans* (Goldman et al. 2004, Li et al. 2004; Xiao et al. 2004). Substitution of Y118 has not been reported but experimentally reported to cause Azole resistance. Y132 has been reported in combination to other mutants (double and quadruplet substitutions occurring simultaneously).

### Heme prosthetic group and the Catalytic cavity of the Wild type ERG 11 of *Candida albicans*

**Figure 6:**
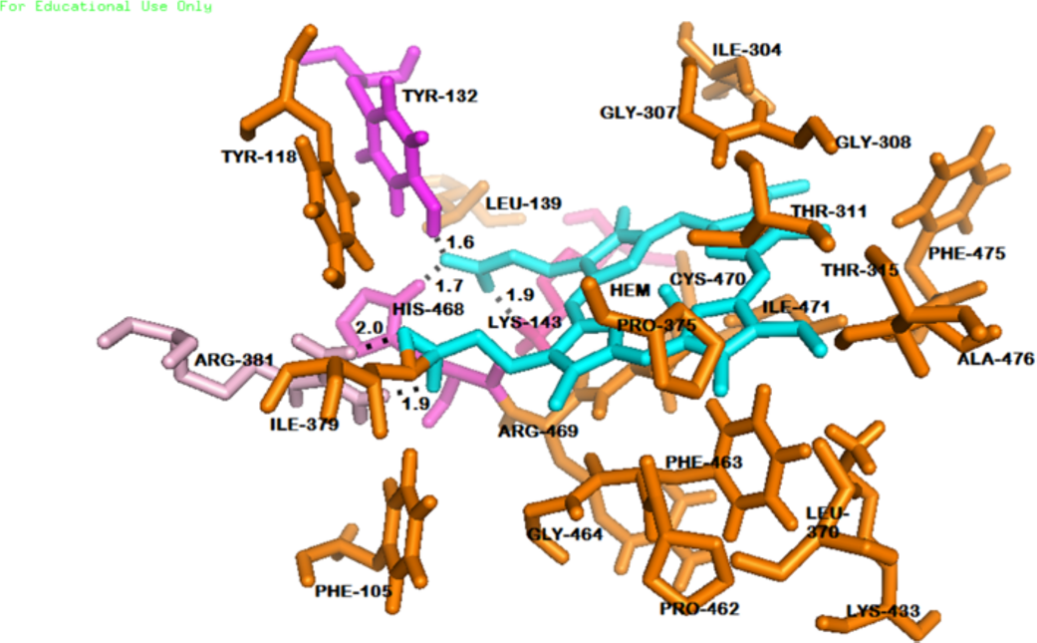
**Amino acid residues within 4 Å from the Heme prosthetic group of wild type ERG11 of** ***C.albicans*** **.**

Five polar contacts are made with four different amino acids (Tyr 132, Lys 143, His 468 and Arg 381) with in 2 Å of the Heme. There are 24 amino acids within 4 Å radius from the prosthetic group (Figure 6). D and A rings of HEME are in close proximity to the ERG11 active site. Polar contacts are made by O1A, O2A and O1D, O2D. C and B rings of Heme are distal to the active site. TYR118 and TYR132 are present within 4 Å of the Heme which are predicted to be vital for catalysis. The catalytic domain is interior (Additional file 1) of the protein (Wild ERG 11) and its dimensions are 1190 Å^3^ (volume) and 1013 Å^2^(Area). Superimposition of the Wild type ERG 11 of *Candida albicans* and the template also revealed the position of the substrate access channels (Figure 5). Opening of these access channels at the entrance of the catalytic furrow are typically flexible as observed in the Normal Mode Analysis of a low frequency mode (mode 7) (Additional file 1).

### Comparative account of wild and the mutant ERG 11 of *Candida albicans*

The single mutants of ERG 11 were screened by direct computational prediction method. A thorough comparative analysis of wild and mutant enzymes were done in terms of the following: (i) active site geometry (volume Å^3^/area Å^2^ of catalytic groove) and topological alterations at the vital region of active site, (ii) comparison of local changes at substitution sites in terms of polar contacts (number/bond length), neighboring residues (4 Å radius) and situation of the Heme (distance in Å from Cα of wild/substituted residue), (iii) comparison of forcefield energetics (GROMOS96) and (iv) of dynamic properties (Mean Square Displacement 〈*R*^2^〉 of lowest-frequency normal mode). Wild and mutant models of *Candida albicans* generated in the study can be acquired from the corresponding author for academic purpose.

### Insight from sequence based computational prediction of the effect of single amino acid substitutions

Mis-sense mutations occurring singly on ERG 11 of *Candida albicans* that are reported to cause Azole resistance (F380S, P230L, Y118A and K342R) were screened by sequenced based methods. A particular single amino acid substitution (mis-sense mutation) was termed ‘Pathogenic’ if all the used classifier predicts it to be deleterious or disease causing. It is observed that K342R mutant is completely non-pathogenic as all the classifier predicts it to be either Neutral or Benign, and it cause a small alteration on quantitative energy parameter DDG value (-0.17 Kcal/mol). Rest of the other single muatnts were significantly ‘Pathogenic’ as all classifier predicted these mutants either “Deleterious” or “Probably Damaging” (Table 3).

**Table 3:** **Sequence based prediction of the effect of single amino acid substitutions on ERG11 of** ***Candida albicans***

Sl.no	Mutant	Classifiers						
		Polyphen		Provean		I-MUTANT		MutPred
		Score	Prediction	Score	Prediction	Effect	DDG value (Kcal/mol)	Probability of deleterious mutation
1	ERG11_D116E	0.003 (sensitivity: 0.98; specificity: 0.44)	Benign	0.695	Neutral	Neutral	-0.47	0.672
	ERG11_Y118A	1.000 (sensitivity: 0.00; specificity: 1.00)	Probably Damaging	-9.487	Deleterious	Large Decrease in stability	-1.83	0.764
2	ERG11_P230L	0.997 (sensitivity: 0.41; specificity: 0.98)	Probably Damaging	-8.773	Deleterious	Large Decrease in stability	-0.97	0.849
3	ERG_F380L	0.831 (sensitivity: 0.95; specificity: 0.82)	Probably Damaging	-3.075	Deleterious	Large Decrease in stability	-1.28	0.583
4	ERG11_K342R	0.005 (sensitivity: 0.97; specificity: 0.74)	Benign	-0.443	Neutral	Neutral	-0.17	0.389

### Observation from four tier comparative biophysical analysis

The two mutants in which the active site dimensions are not changed in spite of amino acid substitution are ERG11_K342R and ERG11_A114S_Y257H, also the mutant ERG11_K342R is significantly more stable than the wild protein. A minimal change in catalytic pocket dimension is observed in ERG11_ D116E while a huge increase in active site dimensions is seen in ERG11_ Y118A. The mutant ERG11_ P230L showed a decrease in active site dimensions. In the rest four mutants there was significant increase of the active site dimension. The quadruplet mutant ERG11_D116E_K128T_Y132H_G465S highest decrease in energetic stability was observed. In the other six mutants there was reduction in energetic stability. The details of comparative energetics of the wild type ERG11 and its mutants are given in the Additional file 1. Also a pictorial description of active site dimensions and the changes brought about at the local environment at molecular level can be found in the Additional file 1. Considering the Mean Square Displacement 〈*R*^2^〉 of wild ERG11 as reference the *R*^2^ plot of the other respective mutants shows that there has been alteration of 〈*R*^2^〉 of the amino acids at various domains. Minimal fluctuations were recorded for the mutants ERG11_ A114S_Y257H and ERG11_K342R (Additional file 1).

### ERG11 phylogeny

**Figure 7:**
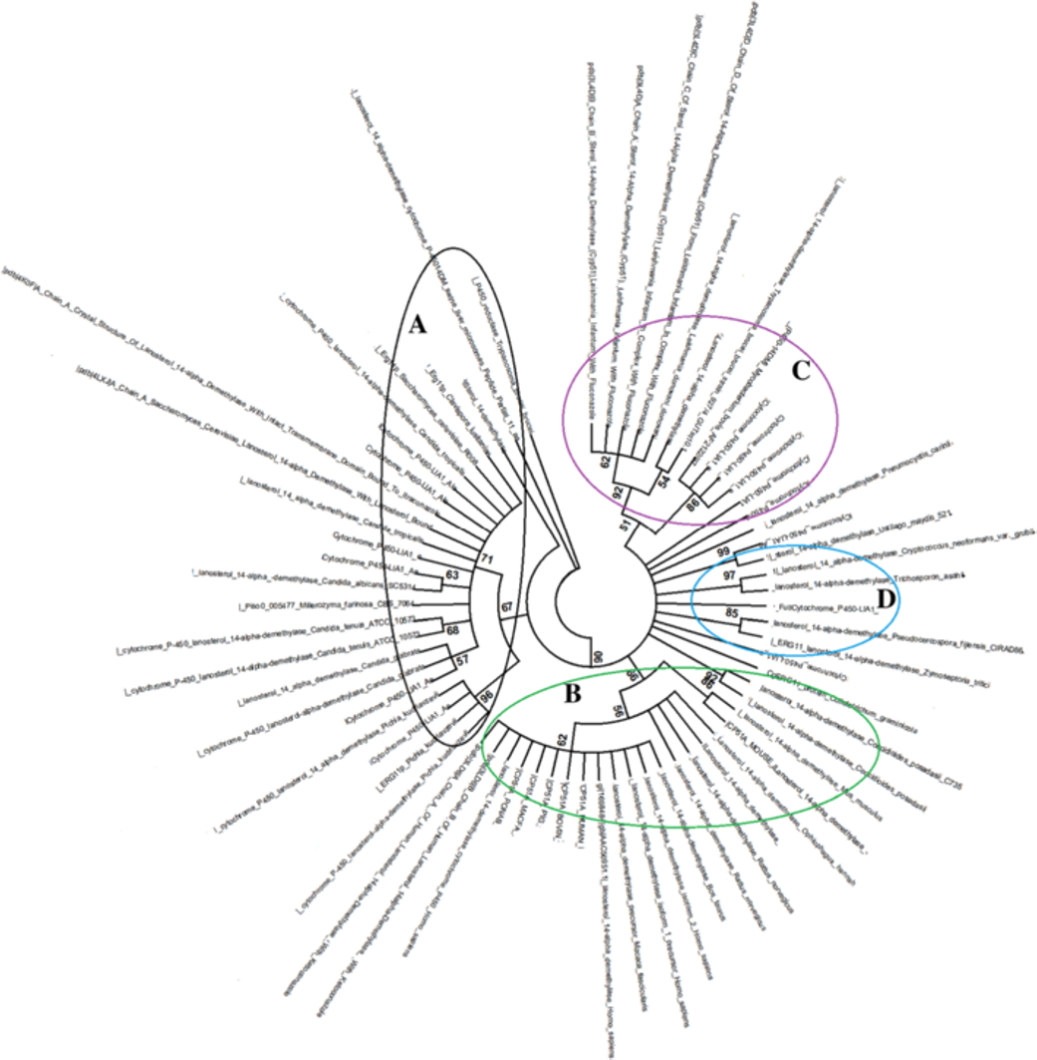
**Phylogeny of ERG11.** Fungal and protozoal Cytochromes or ERG11 are in clad A, the higher animals and the sequences of 3D models (from PDB) are clustered as a clad B and C respectively. Clad D is highly diverse sequences.

For the used dataset LG + G model (Gamma distributed) of substitution was found to be most suitable with lowest BIC score of 2258. 252 and AICc score of 1580.854. The final dataset had 68 protein sequences (Figure 7). The bootstrapped phylogenetic tree (condensed at 50%) had four distinct clads. While all the fungal and protozoal Cytochromes/ERG11 are in clad A, the higher animals and the sequences of 3D models (from PDB) are clustered as a clad B and C respectively. Some highly diverse sequences are clustered together as clad D.

## Discussion

Structural changes in ERG11 are very much likely (Morio et al. 2010) which is evident from the large number and variety of mutations. These mutations are dispersed in hot spots ranging from amino acids 105 to 165, 266 to 287, and 405 to 488 (Marichal et al. 1999). This may indicate that ERG11 is on the verge of a complete restructuring drive. However, in this study it is observed that frequency of mutation is significantly low on the anterior portion of the 3-D catalytic cleft. According to the prediction 14 vital residues are there at this anterior part of the catalytic cleft (Figure 5) with highest number of interactions to a ligand. Surprisingly these residues have the lowest report to have substituted in clinically isolated mutants of *C.albicans*. Only three amino acid residues among the 14 vital residues are found to be substituted in mutant ERG11 of *Candida albicans* (Table 4). All three mutations are associated with confirmed Azole resistance in clinical isolates. Also these are mutations that results in most significant alteration of the active site in terms of all the parameters analyzed in this computational study. These mutations are also less frequent which is evident from scanty number of studies that reports their occurrence. Substitution of Y118 has been predicted to be associated with resistance by in-vitro studies (Chen et al. 2007; Lamb et al. 1997) but has never been recovered in clinical isolates. Similar is the case with the amino acids in the proximity (within 4 Å) of the Heme prosthetic group. Among 24 amino acid residue around the Heme only six amino acids have been found to be substituted in clinical isolates of *Candida albicans* (Additional file 1)*.* All the mutants are exclusively Azole resistant and some are reported to increase Azole MIC by several folds. Mutations in T315 and Y118 have been clearly associated with resistance but not yet detected in clinical isolates (Chen et al. 2007; Lamb et al. 1997). Therefore the observations indicate that a substitution on a vital residue or on a residue close to Heme prosthetic group is less likely to occur. And if at all it occurs will confer strong Azole resistance.

**Table 4:** **Selected mutations on** ***Candida albicans*** **ERG11 and their features**

Sl.no	Mutant	Substitution type	References	Comment	Functional nature substituted residues	
					Active site residues	Vital residues
1	ERG11_A114S _Y257H	Double mutant	Xu et al. 2008; Ge et al. 2010; Ying et al. 2013	Detected in clinical isolates. Described in Azole resistant as well as susceptible isolates. Also described as single mutations (A114S/Y257H) and with various combinations.	None	None
2	ERG11_D116E	Single mutant	Chau et al. 2004; Favre et al. 1999; Marichal et al. 1999; Perea et al. 2001; Sanglard et al. 1998a, 1998b; White et al. 2002	Detected in clinical isolates. Described in Azole-susceptible and Azole-resistant isolates. Also in combination with other mutations.	D116	None
3	ERG11_D116E_K128T_Y132H_G465S	Quadruplet mutant	Ying et al. 2013	Detected in clinical isolates. Detected in Azole resistant strains. Several mutations detected as single mutants.	D116	Y132
4	ERG11_Y118A	Single mutant	Chen et al. 2007; Lamb et al. 1997.	The mutation have been clearly associated with resistance by experimental methods but have not yet been detected in clinical isolates	_	Y118
5	ERG11_Y132H_G450E	Double mutant	Chau et al. 2004; Favre et al. 1999; Goldman et al. 2004; Loffler et al. 1997, Perea et al. 2001	Detected in clinical isolates. Detected only in Azole resistant strains.	_	Y132
6	ERG11_P230L	Single mutant	Li et al. 2004; Xiao et al. 2004	Detected in clinical isolates. Detected only in Azole resistant strains with increased resistance.	_	P230
7	ERG_F380L	Single mutant	Sanglard and Bille, 2002	Detected in clinical isolates. Associated with Azole resistance.	_	F380
8	ERG11_K342R	Single mutant	Goldman et al. 2004, Ying et al. 2013	Detected in clinical isolates. Described only in Azole-susceptible isolate.	None	None

Mutants from N terminal end of ERG11.

In this study single mutations occurring on three types of residues; predicted vital residues, active site residues and non active site residues were included. Initial screening of sequence based computational methods was consistent in sorting the mutants (Table 3) in various categories of severity. ERG11_Y118A was the most “Pathogenic” followed by ERG11_ F380S and ERG11_ P230L. The mutant ERG11_ D116E was intermediate in terms of pathogenecity and ERG11_K342R was completely neutral. The geometry of the catalytic pocket was found to be significantly altered in the mutants ERG11_ Y118A, ERG11_ F380S and ERG11_ P230L. An increase in volume and area of the catalytic pocket in ERG11_ Y118A and ERG11_ F380S may lead to decreased affinity of Azole. Similarly a decrease in volume and area (in ERG11_ P230L) may lead to inaccessibility of catalytic site and subsequent resistance. This may explain exclusive Azole resistance in the clinical isolates of ERG11_ F380S and ERG11_ P230L. Clinical isolates of ERG11_Y118A are not reported and this indicates the functional significance of the residue Y118. Geometrical changes at the catalytic pocket in the mutants may have occurred due to significant changes in the local environment, for example in ERG11_Y118A, polar contacts with neibouring vital residues were demolished and the distance of prosthetic group from the mutation site increased. Similarly in ERG11_ F380S vital residues from the vicinity were missing and an increase in polar distance among vital residues may have resulted in geometrical distortion of active site. In ERG11_P230L, there has been a change in the local rigidity, a parameter that may be important in functional folds of an active site (Charbonnier et al. 1999) as Proline has been substituted. A Proline substitution may change the immediate chemical surrounding of a nucleophile by making it more hydrophilic (Roos et al. 2007). The specific conformation of Proline imposes many restrictions on the structural aspects of peptides and proteins conferring particular biological properties (Cunningham and O’Connor, 1997). The alteration of geometry due to the vital residue substitutions in the mutants by means of surface generation is shown in the Additional file 1. In ERG11_D116E and ERG11_K342R there is either minimal or no change in the active site geometry. Since ERG11_K342R is a distant residue therefore the local changes taken place in the mutant have not influenced the active site. The minimal change on active site in ERG11_D116E can be attributed to the the substitution of a hydrophilic and negatively charged residue by another similar amino acid, however Glutamate is slightly bigger than Aspartate. It is also observed that the Energy scores (GROMOS97) of the mutants can indicate the consequences of the mutations. Among the single mutations F380L is the most destabilizing followed by Y118A and P230L. On the other hand K342R mutation is highly stabilizing which shows minimum fluctuations in the Normal Mode Analysis. The mutant ERG11_D116E is intermediate in terms of energy scores. Among the clinical isolates harboring single mutations, considerable diversity in resistant phenotype is observed. For example ERG11_K342R is isolated exclusively from susceptible strains while ERG11_D116E was isolated from resistant as well as susceptible *C.albicans*. From the analysis of single mutants it is assumed that vitality of an amino acid for possible interactions with a ligand at the active site is an important factor that determines severity of active site alteration on the occurrence of a mutation leading to Azole resistance in *Candida albicans*.

Three mutants in which multiple mutations coexist on the ERG11 were analyzed. In the mutant ERG11_A114S_Y257H, none of the substituted residues were vital residues or active site residues. The mutations did not alter any local parameters and had no effect on the active site geometry, which explains its occurrence in susceptible isolates. But interestingly the mutant is also isolated from Azole resistant strains as well. A possible explanation for occurrence of this double mutant in susceptible as well as resistant clinical isolates may be harboring of other unrelated mutations (such as on the efflux pump proteins) in the strain that results in resistant phenotype. It may be noted that occurrence of multiple mutations in ERG11 may not ensure alteration in catalytic pocket leading to confirmed resistance in clinical isolates. In the double mutant ERG11_Y132H_G450E significant alteration of the local environment has lead to severe alteration of the active site. This may explain its occurrence on only resistant strains and also indicate that the vitality of Y132 is indispensable for ERG11 geometry. The double mutations Y132H, G450E on the ERG11 is also considerably more destabilizing then the double mutations A114S, Y257H. Dynamic changes on the mutants as explained by Normal Mode Analysis also indicate significantly more alteration in ERG11_Y132H_G450E than that of the ERG11_A114S_Y257H. The quadruplet mutant ERG11_D116E_K128T_Y132H_G465S has been isolated in fungus with reduced susceptibility to Azole. Single mutant with D116E resulted in minimal alteration of active site and another double mutant with Y132H resulted in significant change. In this quadruplet mutant alteration of active site is more than the both of the single or double mutant. This mutant structure is also most unstable in energetic terms and posse’s significant fluctuation of its molecular motions.

## Conclusion

It is apparent that significant geometrical changes in the ERG11 active site domain will take place if the amino acids responsible for key molecular interactions (with its substrate or inhibitors) are substituted. An increase or decrease in the catalytic pocket dimension is most likely to bring about Azole resistance. Several parameters of the local environment determine the severity of the change in active site dimension. This study identifies the vital residues, mutations of which will cause confirmed Azole resistance. If a mutation is far from the active site or the Heme group it may not cause Azole resistance. Moreover, Azole resistance in *Candida albicans* may be a multifactorial process (Balkis et al. 2002) that can be mediated through multiple underlying mechanisms independent of ERG11. Resistance can be acquired through (i) transcriptional activation leading to over expression of the genes encoding at least two groups of efflux pumps either belonging to ABC (the ATP-binding cassette that include CDR1, CDR2 etc.) or MFS (major facilitator include MDR1) super-families of proteins (White 1997a, 1997b; Maebashi et al. 2002). This results in reduced intracellular accumulation of drugs.(ii) Altering the synthetic pathway of fungal sterols by-passing ERG11 (Claire et al. 2010) is also reported as a mechanism of resistance. (iii) The fungus may even induce chromosomal aneuploidy (Perepnikhatka et al. 1999; Selmecki et al. 2009) as chromosome 5 duplication or the presence of a chr5L isochromosome to acquire resistance. It is also important to note that, several of these mechanisms are often found to be combined in clinical isolates (Cernicka and Subik, 2006; Chau et al. 2004; Coste et al. 2007; Franz et al. 1998; Goldman et al. 2004). Therefore in order to device a full proof strategy to combat Azole resistance functional genomics has to explore all possible drug targets.

This analysis showed that substitution of amino acids that posses maximum interaction with substrate or inhibitor at the ERG11 active site have highest propensity to cause confirmed resistance by geometrical change of the catalytic pocket. Intra residue local interaction among amino acids of the catalytic site may be a determining factor if it involves one or more vital residues. But if the substituted residue is far from the anterior part of the ERG11 active site or at a distant location from Heme group, it may not determine Azole resistance by geometrical change of the active site. However, since a large amount of mutations are yet to be explained for their Azole resistance it is argued that other underlying mechanisms (as reviewed by Prasad and Kapoor 2004) may be involved. For effective therapeutic intervention against resistant *C.albicans* in T.B or AIDS patients novel drug targets may be prioritized. The mutant models used in the study may be used for further insight of Azole binding in the active site or to analyze potential interaction with the Heme prosthetic group via methods like Quantom Mechanics-Polarized Ligand Docking (QPLD). This computational study may be considered a dry-run prior to the wet-lab experimentations that involve significant investment in various terms. The *in-silico* strategy to identify vital residues on *C.albicans* ERG11 can substantially speed up experimental work and reduce associated costs towards characterization of mutations.

## Materials and method

In this study the objective is to evaluate (i) how Azole resistance in *C.albicans* can be accounted for structural changes in the ERG11 active site. The paper evaluated (ii) the role of local environment (polar bonds, proximal residues and Heme prosthetic group) around amino acid substitutions. Analysis of mutation induced alterations in (iii) energetic stability in 3D models and (iv) dynamic changes (by normal Mode Analysis) were done. ERG11 phylogeny analysis will throw light on its variability among several taxa.

### Primary sequence of *Candida albicans*ERG11

Uniprot, a global database was queried for Lanosterol 14-alpha-Demethylase (ERG11) in *Candida albicans*. A reviewed entry for ERG11 in *C.albicans*, the NCBI Reference Sequence: XP_716761.1 of *C.albicans*, strain SC5314 was selected for the study. The primary sequence is downloaded in FASTA format and used as the wild type ERG11 sequence. Composition of amino acids in ERG11 and its physicochemical parameters were computed (Protparam).

### Homology Modeling of *Candida albicans*ERG11

Out of several million protein sequences, less than 0.1 million have their structures determined experimentally by X-ray or NMR. However, increased reliability of alignment algorithms and modeling programs paved a way to predict 3D coordinates of proteins for which we only have knowledge of the primary sequence sharing certain homology with that of experimentally determined proteins.

Swiss-model (Kiefer et al. 2009) is a user friendly and computationally economic method that generates several perspective models of the query and ranks them based on a scoring scheme customized in the algorithm. It uses various templates and analyses them in terms of QMEAN Z-Score. The higher is the score; the better is the model quality. It also describes the modeled residue range, the template information with its resolution, percentage of sequence identity as well as a E-value. According to the need the user can also specify a PDB template by its code or can upload a PDB file to be used as a template. Most importantly, a predicted structure from the algorithm accommodates the co-ligands or metal atoms, presence of which (co-ligands and metal ions) may determine structural or functional integrity of the protein.

### Energetic refinement and geometrical validation of models

The overall stereochemical quality of the protein models were assessed and Molprobity (Chen et al. 2009) provides broad spectrum solidly based evaluation of model quality at both global and local levels. It utilizes the power and sensitivity provided by optimized hydrogen placement and all-atom contact analysis, complemented by updated versions of covalent geometry and torsion angle criteria. Molprobity generates all atoms Clashscore which is the number of serious steric overlaps (>0.4 Å) per 1000 atoms. The classical Ramachandran plot analysis is done in-terms of the number and percentage of Ramachandran outliers and Ramachandran favored residues. The Molprobity score, is a protein geometry parameter that is dependent upon the Ramachandran outliers and favored residue percentage. It also gives the number and percentage of Poor roamers, Cβ deviations >0.25 Å, Bad backbone bonds and angles.

Stereochemical quality of reconstructed protein models are conventionally enhanced by energy minimization protocols. This improves the physical realism and structural accuracy of protein models. Accuracy in protein structure is improved by repairing distorted geometries if any, for example by removing steric clashes (Chou and Carlacci, 1991) and by curtailing the free energy to make the protein stable. SwissPDB-Viewer, SPdbV (Guex, and Peitsch, 1997) minimizes energy of 3D structures implementing GROMOS96 forcefield and the computations are done in vacuum devoid of a reaction field. User can optimize the steps of conjugate gradient or steepest descent methodologies. The software returns energy scores in terms of bond, non-bond, angles, torsion, improper and electrostatic energies in Kilo Jules/mole. The user can program the energy optimization cycles to stop when ΔE between two subsequent steps or even the force acting on any atom of the 3D model is below a threshold. The user can either use a harmonic constraint or can lock on selected residues for energy optimization.

### Optimization of model quality

Homology modeling although uses an experimentally determined PDB template, the resultant model may not achieve finer structural quality. The optimization protocol assumes that the values of quality assessment parameters of a good homology model should approach towards similar assessment values of the template (experimentally determined 3D co-ordinates). For this the model as well as the template (used for homology modeling) are analyzed by Molprobity in terms of Clashscore, Poor rotamers, Ramachandran outliers and favored, MolProbity score, Cβ deviations, Bad backbone bonds and angles. Molprobity parameters of the template were considered as the benchmark value of protein quality for a respective model. Energy minimization of the models by executing GROMOS96 force field method was done in steps of 20 cycles of Steepest descent and/or Conjugate gradient. After every step of 20 cycles the quality was again assessed by Molprobity. Cycles of minimization were carried out until the quality approaches the benchmark values of the template. If quality of model deteriorates, minimization steps were stopped. *Candida albicans* ERG 11 Model with best quality parameters was selected for further analysis.

### Analysis of active site characteristics of *Candida albicans*ERG 11: Identification of vital residues in the active site by composite approach

It is assumed that higher the number of atomic interactions upon an amino acid residue, higher is its probability to be imperative in the active site chemistry. Vital amino acids in the 3D active site of *Candida albicans* ERG 11 were identified by a composite approach via Q-SiteFinder (Laurie and Jackson 2005) of the Leeds University server. It uses the van der Waals interaction of a methyl probe and an interaction energy threshold to determine favorable binding clefts and not solely depend on geometrical criteria for prediction. The output lists all the potential atomic interactions incurred by the amino acids. Thus multiple interactions upon a single amino acid residue are predicted. Since this application probes site binding energies with the appropriate energy cut-off rather than purely geometric criteria to determine favorable binding sites on proteins, it efficiently reduces the tendency to increase predicted site volumes with protein size.

The composite approach for predicting vital active site residues in *Candida albicans* ERG 11 followed these steps: (i) initially all the active site residues were predicted from optimized and validated homology model of *Candida albicans* ERG 11 as well as from the PDB template (experimentally determined 3D protein structure from RCSB PDB database). (ii) Predicted active site residues with the number of atomic interactions upon each of the amino acids (both for model and template) were noted. (iii) From each protein (model and template) the amino acid with highest number of interactions and amino acids with interactions at least half of that highest number were identified. These amino acids are assumed to be the most potential candidates for ligand binding at the active site. (iv) Primary sequences of the *Candida albicans* ERG11 homology model and its template (in FASTA format) were aligned by the Needle program of EMBL. (v) Mapped of the potential active site residues of the template were done upon the *Candida albicans* ERG 11 homology model from the alignment. Finally (vi) the Vital active site residues of *Candida albicans* ERG 11 were assigned by compositing predictions of potential amino acids for the model as well as the template.

The template which is an experimentally determined crystal structure of a protein may have an already bound substrate or inhibitor co-crystallized at the catalytic cleft. The bound ligand may increase probable prediction of vital residues. Therefore, in the composite search approach for vital residues the template along with the homology model was used. So the definition of Vital amino acid residues on the active site of *Candida albicans* ERG 11 stands as: the active site amino acids with maximum propensity for interaction. The composite approach for detection of vital amino acids at the *Candida albicans* ERG 11 active site was found to be effective as evident from the results.

### Analysis of active site geometry of *Candida albicans*ERG 11: The Substrate access channels, their dynamics and the Heme moiety

In order to visualize the catalytic domain of *Candida albicans* ERG 11 the optimized and validated homology model is superimposed on to the template structure in Pymol (DeLano 2002). The template was visualized in its secondary structure (helix-sheet-loop) and the *Candida albicans* ERG 11 homology model was visualized with each amino acid in its native conformation (stick). The surface of the residues was generated that were predicted to be the vital residues at active site as per the composite approach. The template structure has a bound ligand on its active site, and the superimposition with surface generation of vital active site residues clearly showed the location of the access channels and the active site cavity. This visualization indicated that the composite approach of identification of vital residues at the active site is a suitable method.

Cavities in proteins may generally be related with the dynamics and function of a protein since it accommodates a substrate. Thermodynamic and mechanical properties of catalytic proteins which accommodate a substrate in its interior may be such that the channel gates of its catalytic cleft will vibrate in a Normal mode. Normal mode analysis characterizes the collective motions of a group of atoms which is based on the harmonic approximation of the potential energy function around a minimum energy conformation (Suhre and Sanejouand, 2004). Therefore, normal mode calculations provide an alternative to molecular dynamic simulations for studying collective motions in macromolecules (Shahila et al. 2003). Analysis of Mean Square Displacement 〈*R*^2^〉 of the lowest-frequency normal mode to study the dynamic behavior of *Candida albicans* ERG 11 homology model was done (ElNe’mo, Suhre and Sanejouand, 2004).

The mechanically flexible substrate access channels lead to a catalytic cavity and its volume and area determines its substrate or inhibitor binding propensity. Analysis of the dimensions of the *Candida albicans* ERG 11 catalytic pocket in terms of volume (Å^3^) and area (Å^2^) was done. Local environment of the Heme prosthetic group was analyzed in terms of polar contacts and identification of the neighboring residues within a 4 Å radius around the Heme.

### Missense mutations on ERG 11 of azole resistant *Candida albicans*

Review of literature indicated that missense mutations on ERG 11 of *Candida albicans* may either occur singly or in combination. The dataset of mutant ERG 11 of *Candida albicans* for this study focused on the following: (i) mutants with single and multiple amino acid substitution/s (ii) single mutants with substitution on a predicted vital residues and non-vital residues, (iii) multiple mutants with substitution of at least one predicted vital amino acid residue (with concurrent substitution/s of non vital or active site residue/s), (iv) single and multiple mutants with substitutions only on non vital residue/s. Mutants were (Table 4) identified from literature followed by the prediction of vital active site residues of ERG 11 in *Candida albicans* by means of the composite approach (results section)*.*

### Modeling the mutant ERG 11 proteins of *Candida albicans*

The optimized and validated homology model of wild type *Candida albicans* ERG 11 was used for modeling the mutant ERG 11 of Azole resistant *Candida albicans* by site specific substitution. Wild type amino acids were substituted by the mutant residues for the single, double and quadruplet amino acids. For every substitution SPdbV generates several conformations. The conformation with highest energetic stability was selected for each substitution. This ensures uniformity in terms of energetics during the amino acid substitutions. All the mutant models were further optimized and validated following the method explained for optimization and validation of the wild ERG11 model.

### Direct sequence based computational prediction of the effect of single amino acid substitutions

Computational analysis to study role of a mutation may be used for screening purpose prior to a computationally expensive study.

### Evolutionary trend based analysis

Polymorphism Phenotyping (PolyPhen, Adzhubei et al. 2010) and Provean (Choi 2012) are evolutionary trend based classifier of amino acid substitutions. PolyPhen utilizes a combination of sequence and structure-based attributes for the description of an amino acid substitution, and the effect of mutation is predicted by a native Bayesian classifier. Provean runs a Blast search and the clustering of BLAST hits is performed by CD-HIT with a parameter of 75% global sequence identity. The top 30 clusters of closely related sequences form the supporting sequence set are used to generate the prediction.

### Analysis based on free energy change

Amino acid substitutions leading to alteration of stability in *Candida albicans* ERG 11 were predicted via a neural-network-based application. The tool (I-Mutant) was trained on a data set derived from ProTherm (Bava et al. 2004), a comprehensive database of protein mutations.

### Molecular effect based prediction

Mutations effect protein functions at molecular level and an algorithm (MutPred, Li et al. 2009) trained using the deleterious mutations from the Human Gene Mutation Database (Stenson et al. 2009) and neutral polymorphisms from Swiss-Prot (Boeckmann et al. 2003) efficiently classifies them in *Candida albicans* ERG 11.

### Biophysical analysis of *Candida albicans*ERG 11 and its mutants

Amino acid substitution in a catalytic protein may alter several of its characteristic properties leading to alteration in its 3D active site. In this section several biophysical parameters for a comparative account among the wild and mutant *Candida albicans* ERG 11 were considered.

### Comparison of active site geometry of *Candida albicans*ERG 11 and its mutants

The volume and the area of the catalytic cleft are important factor as this accommodates a substrate or a substrate inhibitor. Analysis of the volume (Å^3^) and the area (Å^2^) of the ERG 11 active site in the wild type and the mutant proteins was done. Cavity detection algorithm (SPdbV) identifies potential cavities on a protein and returns the volume and area. The geometrical parameters were noted for each model and compared with the wild type parameters.

### Comparative analysis of local environmental changes at the amino acid substitution sites in the *Candida albicans*ERG 11 and its mutants

Influence of an amino acid on its local environment can be analyzed in terms of its interaction with other residues. Substitution of the amino acid may or may not alter the interactions of the wild type residue in the polypeptide chain. This in turn may influence structure and function. Number of polar interactions and the bond lengths are measured in the wild type and mutant ERG 11 at the wild and substituted amino acids (Pymol). The neighboring residues were identified at wild and substituted sites with in a 4 Å radius from the Cα of the amino acids.

The distance of the Heme prosthetic group (in Å) was determined from the wild and mutant residues in the proteins. This analysis may indicate the influence of the missense mutations locally on the protein phenotype. Local environment of the mutant in comparison to the wild residue on the basis of amino acid characteristic were also analysed (HOPE, Venselaar et al. 2010).

### Comparison of forcefield energetics of *Candida albicans*ERG 11 and its mutants

Molecular mechanics or force field methods use classical type models to predict the energy of the molecule as a function of its conformation. This allows prediction of equilibrium geometries, transition states and relative energies between conformers or between different molecules. The total energy is expressed as a sum of Taylor series expansions for the stretches for every pair of bonded atoms, and adds additional potential energy terms contributed by bending, torsional energy, van der Walls energy, and electrostatics (Leach, 2001). Energy scores in terms of bond, non-bond, angles, torsion, improper,electrostatic energies and the total energy of the models in Kilo Jules/mole were estimated (GROMOS96). The reference energy values of wild *Candida albicans* ERG 11 was compared with energy values of the mutants.

### Comparison of dynamic properties of *Candida albicans*ERG 11 and its mutants

Mean Square Displacement 〈*R*^2^〉 was calculated for all protein models of ERG 11. The *R*^2^ values of wildtype protein are the reference against which mutant values are comparatively plotted. A straight line in the plot for a protein will indicate complete identity of molecular motion with the reference protein, wild ERG11. The comparative analysis may indicate dynamic changes brought about by the mis-sense mutations.

### Phylogenetic analysis of ERG11

Protein sequences (FASTA) from GenBank were retrieved. Out of 421 entries the hypothetical proteins, duplicate entries, un-named proteins and vitamin D hydroxylases were removed. Selected protein sequences were aligned by ClustalW (Blosum Protein Weight Matrix) and several duplicate entries were removed by default. Most suitable substitution model was identified using maximum likelihood statistical method with complete deletion and very strong branch swap filter (MEGA 6, Tamura et al. 2013). Phylogeny reconstruction was done using the identified substitution model and Maximum Likelihood (ML) statistical method with Nearest-Neighbor-Interchange (NNI) heuristic method. Gaps or missing data were completely deleted to increase robustness. Bootstrap test of phylogeny was done with 500 replications. The final tree was condensed at 50% cut-off value.

## Electronic supplementary material

Additional file 1: **Mis-sense mutations only on some vital residues of Lanosterol 14-alpha–demethylase (ERG11p) can be traced to Azole resistance in Candida albicans clinical isolates.** (DOCX 4 MB)

## Abbreviations

ERG11: 14a-Lanosterol demethylase of ergosterol synthesis pathway
NMR: Nuclear magnetic resonance spectroscopy
FASTA: FAST alignment
KEGG: Kyoto encyclopedia of genes and genomes
RMSD: Root mean square deviation
MIC: Minimal inhibitory concentration
Å: Angstrom
AICc score: Akaike information criterion score
BIC: Bayesian information criterion
Da: Dalton
FLZ: Fluconazole
KJ: Kilo Jules
PDB: Protein data bank
NCBI: National Centre for biotechnology Information.
