# Supplementary material for: Structural basis for heterogeneous phenotype of ERG11 dependent Azole resistance in C.albicans clinical isolates
Source: Springerplus. 2014 Nov 6;3:660. doi: 10.1186/2193-1801-3-660 (PMC4237678; doi:10.1186/2193-1801-3-660)
Supplement: Supplementary file 1 — Additional file 1: Mis-sense mutations only on some vital residues of Lanosterol 14-alpha–demethylase (ERG11p) can be traced to Azole resistance in Candida albicans clinical isolates. (DOCX 4 MB) [file 40064_2014_1366_MOESM1_ESM.docx]

**Additional file 1**

**Mis-sense mutations only on some vital residues of Lanosterol 14-alpha–demethylase (ERG11p) can be traced to Azole resistance in *Candida albicans* clinical isolates**

**Surajit Debnath 1 and Soma Addya 2**

**1 Department of Medical Laboratory Technology, Women’s Polytechnic, Hapania,Tripura (W), surajit03@yahoo.co.in**

1. **General physician, Paschim Medinipur, West Bengal**

**Quality improvement regime for *C.albicans* wild type ERG11 model**

The combination of structure optimization cycles for the ERG11 model followed: 20 cycles of steepest descent (SD) , 20 cycles of conjugate gradient (CG), 20 cycles of SD/20 cycles of CG , 40 cycles of SD , 40 cycles of CG , 40 cycles of SD/40 cycles of CG.

In the initial ERG11 model (TABLE S1a) there were five poor rotamers (1.08%) , 490 Ramachandran Favoured residues (94.59%) and there were eight (1.64%) Cβ deviations (>0.25Å). In the template structure (TABLE S1b) the percentage of Ramachandran Favored residues were significantly more (96.98%). Initial total energy score for homology model was -20340.652Kilo Jules/mole.

Continual quality assessment (Molprobity) of models (TABLES S1c – S1e) showed optimum results with 40 cycles of steepest descent .The final model quality (TABLE S1c) approached the benchmark values of the template.

**Table S 1a : Quality assessment of the initial model of ERG11 *Candida albicans*.**

| All-Atom Contacts | Clashscore, all atoms: | 29.17 | | 16^th^ percentile^*^ (N=1784, all resolutions) |
| --- | --- | --- | --- | --- |
|  | Clashscore is the number of serious steric overlaps (> 0.4 Å) per 1000 atoms. | | | |
| Protein Geometry | Poor rotamers | 5 | 1.08% | Goal: <1% |
|  | Ramachandran outliers | 5 | 0.97% | Goal: <0.05% |
|  | Ramachandran favored | 490 | 94.59% | Goal: >98% |
|  | MolProbity score^^^ | 2.35 | | 56^th^ percentile^*^ (N=27675, 0Å - 99Å) |
|  | Cβ deviations >0.25Å | 8 | 1.64% | Goal: 0 |
|  | Bad backbone bonds: | 30 / 4345 | 0.69% | Goal: 0% |
|  | Bad backbone angles: | 21 / 5894 | 0.36% | Goal: <0.1% |

|  |
| --- |

| \| **Table S1 b: Quality assessment of the template 4k0f.pdb used for modeling of *Candida albicans* ERG11.It is the benchmark of model quality.**   \| All-Atom Contacts \| Clashscore, all atoms: \| 4.25 \| \| 99^th^ percentile^*^ (N=481, 2.19Å ± 0.25Å) \| \| --- \| --- \| --- \| --- \| --- \| \| Clashscore is the number of serious steric overlaps (> 0.4 Å) per 1000 atoms. \| \| \| \| \| Protein Geometry \| Poor rotamers \| 11 \| 2.36% \| Goal: <1% \| \| Ramachandran outliers \| 1 \| 0.19% \| Goal: <0.05% \| \| Ramachandran favored \| 514 \| 96.98% \| Goal: >98% \| \| MolProbity score^^^ \| 1.66 \| \| 96^th^ percentile^*^ (N=10466, 2.19Å ± 0.25Å) \| \| Cβ deviations >0.25Å \| 0 \| 0.00% \| Goal: 0 \| \| Bad backbone bonds: \| 0 / 4476 \| 0.00% \| Goal: 0% \| \| Bad backbone angles: \| 0 / 6080 \| 0.00% \| Goal: <0.1% \| \| \| --- \| --- \| --- \| --- \| --- \| --- \| --- \| --- \| --- \| --- \| --- \| --- \| --- \| --- \| --- \| --- \| --- \| --- \| --- \| --- \| --- \| --- \| --- \| --- \| --- \| --- \| --- \| --- \| --- \| --- \| --- \| --- \| --- \| --- \| --- \| --- \| --- \| --- \| --- \| |
| --- | --- | --- | --- | --- | --- | --- | --- | --- | --- | --- | --- | --- | --- | --- | --- | --- | --- | --- | --- | --- | --- | --- | --- | --- | --- | --- | --- | --- | --- | --- | --- | --- | --- | --- | --- | --- | --- | --- | --- |
| **Table S1 c : Quality assessment of ERG11 *Candida albicans* model after 40 cycles of Steepest Descent in GROMOS97 .**   \| All-Atom Contacts \| Clashscore, all atoms: \| 14.61 \| \| 51^st^ percentile^*^ (N=1784, all resolutions) \| \| --- \| --- \| --- \| --- \| --- \| \| Clashscore is the number of serious steric overlaps (> 0.4 Å) per 1000 atoms. \| \| \| \| \| Protein Geometry \| Poor rotamers \| 4 \| 0.92% \| Goal: <1% \| \| Ramachandran outliers \| 5 \| 0.97% \| Goal: <0.05% \| \| Ramachandran favored \| 493 \| 95.17% \| Goal: >98% \| \| MolProbity score^^^ \| 2.01 \| \| 75^th^ percentile^*^ (N=27675, 0Å - 99Å) \| \| Cβ deviations >0.25Å \| 5 \| 1.02% \| Goal: 0 \| \| Bad backbone bonds: \| 23 / 4345 \| 0.53% \| Goal: 0% \| \| Bad backbone angles: \| 15 / 5894 \| 0.25% \| Goal: <0.1% \| |

|  |
| --- |
| **Table S1 d : Quality assessment of ERG11 *Candida albicans* model after 40 cycles of Steepest Descent and 20 cycles of Conjugate Gradient in GROMOS97 .**   \| All-Atom Contacts \| Clashscore, all atoms: \| 12.2 \| \| 62^nd^ percentile^*^ (N=1784, all resolutions) \| \| --- \| --- \| --- \| --- \| --- \| \| Clashscore is the number of serious steric overlaps (> 0.4 Å) per 1000 atoms. \| \| \| \| \| Protein Geometry \| Poor rotamers \| 4 \| 0.92% \| Goal: <1% \| \| Ramachandran outliers \| 5 \| 0.97% \| Goal: <0.05% \| \| Ramachandran favored \| 490 \| 94.59% \| Goal: >98% \| \| MolProbity score^^^ \| 1.97 \| \| 77^th^ percentile^*^ (N=27675, 0Å - 99Å) \| \| Cβ deviations >0.25Å \| 6 \| 1.23% \| Goal: 0 \| \| Bad backbone bonds: \| 22 / 4345 \| 0.51% \| Goal: 0% \| \| Bad backbone angles: \| 13 / 5894 \| 0.22% \| Goal: <0.1% \| |

|  |
| --- |
| **Table S1 e: Quality assessment of ERG11 *Candida albicans* model after 40 cycles of Conjugate Gradient in GROMOS97.**   \| All-Atom Contacts \| Clashscore, all atoms: \| 14.44 \| \| 52^nd^ percentile^*^ (N=1784, all resolutions) \| \| --- \| --- \| --- \| --- \| --- \| \| Clashscore is the number of serious steric overlaps (> 0.4 Å) per 1000 atoms. \| \| \| \| \| Protein Geometry \| Poor rotamers \| 4 \| 0.92% \| Goal: <1% \| \| Ramachandran outliers \| 5 \| 0.97% \| Goal: <0.05% \| \| Ramachandran favored \| 492 \| 94.98% \| Goal: >98% \| \| MolProbity score^^^ \| 2.01 \| \| 75^th^ percentile^*^ (N=27675, 0Å - 99Å) \| \| Cβ deviations >0.25Å \| 7 \| 1.43% \| Goal: 0 \| \| Bad backbone bonds: \| 24 / 4345 \| 0.55% \| Goal: 0% \| \| Bad backbone angles: \| 18 / 5894 \| 0.31% \| Goal: <0.1% \| |

**Comparative energetics of the wild type ERG11 and its mutants**

Wild ERG11 and the eight different mutants of ERG11 were compared on the basis of the energy scores. The results are tabulated below.

**Table S 2 : Comparative analysis of Energy values among the mutants and wild ERG11 of *Candida albicans***

| **Sl No** | **Proteins** | **Energy Parameters** | | | | | | |
| --- | --- | --- | --- | --- | --- | --- | --- | --- |
|  |  | **Bonds Energy (KJ/mole)** | **Angels**  **Energy (KJ/mole)** | **Torsion**  **Energy (KJ/mole)** | **Improper**  **Energy (KJ/mole)** | **Nonbonded**  **Energy (KJ/mole)** | **Electrostatic**  **Energy (KJ/mole)** | **Toal**  **Energy (KJ/mole)** |
| **1** | **Wild ERG11** | 439.711 | 3200.458 | 2128.688 | 657.486 | -16825.04 | -14511.09 | -24909.789 |
| **2** | **ERG11_ A114S_Y257H** | 451.811 | 3199.049 | 2129.274 | 654.708 | -16646.23 | -14494.02 | -24705.412 |
| **3** | **ERG11_D116E** | 440.784 | 3200.329 | 2129.938 | 657.374 | -16801.37 | -14493.21 | -24866.148 |
| **4** | **ERG11_D116E_K128T_Y132H_G465S** | 452.707 | 3196.537 | 2131.644 | 656.066 | -15896.57 | -14512.67 | -23972.287 |
| **5** | **ERG11_Y118A** | 439.215 | 3193.192 | 2128.274 | 655.478 | -16772.21 | -14457.92 | -24813.973 |
| **6** | **ERG11_Y132H_G450E** | 452.167 | 3197.598 | 2127.746 | 656.207 | -16479.46 | -14558.73 | -24604.473 |
| **7** | **ERG11_P230L** | 439.534 | 3190.085 | 2118.455 | 657.041 | -16724.95 | -14509.17 | -24829.008 |
| **8** | **ERG11_K342R** | 440.532 | 3197.699 | 2122.963 | 657.547 | -16769.10 | -14758.35 | -25108.711 |
| **9** | **ERG_F380S** | 440.357 | 3185.434 | 2125.812 | 655.720 | -16627.10 | -14517.71 | -24737.490 |

**Catalytic pocket of wild type ERG11**

Dimension of the catalytic pocket of wild type ERG11 in terms of volume (Å^3^) and area (Å^2^) is the reference value for evaluation of the effects of the mutations. The wild type parameters are depicted below (Figure S1) :

**
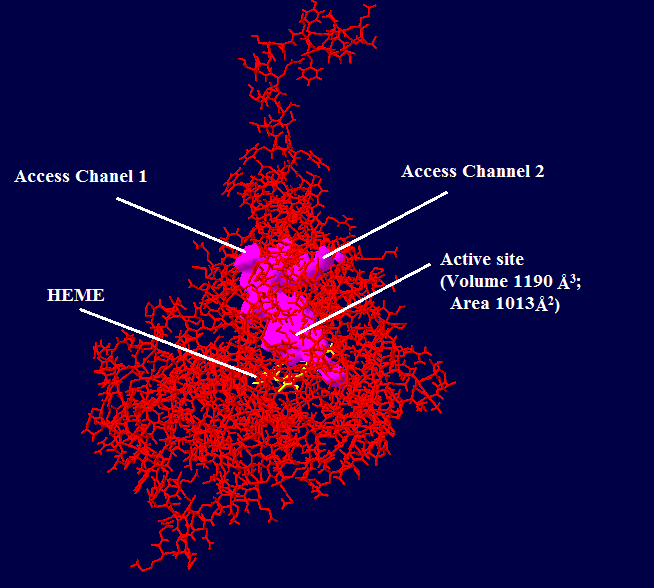
**

**Figure S1 : Wild type ERG11 of *C.albicans* showing the catalytic pocket and substrate access channels. The catalytic domain is interior of the protein. The volume and area of the Wild type ERG 11 catalytic pocket are also shown.**

**Flexibility of the substrate access channels**

Opening of these substrate access channels at the entrance of the catalytic furrow are flexible as observed in the figures.

**
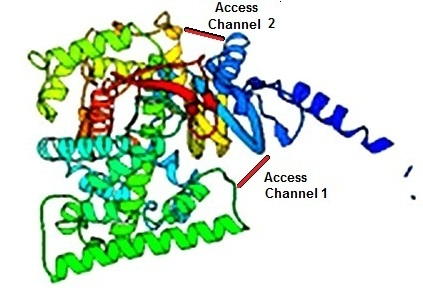

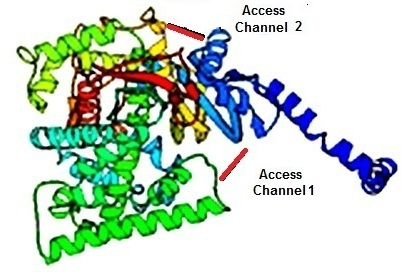

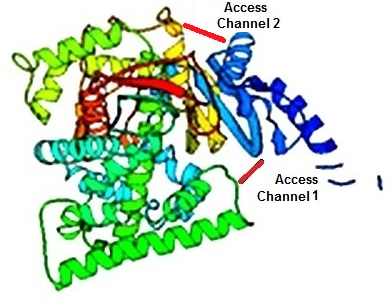
**

**Figure S2 : Flexibility of the substrate access channels in a Low frequency Normal Mode of wild type ERG11 of *C.albicans*.**

**Observation from four tier comparative biophysical analysis**

**Single mutants on ERG 11 of *Candida* *albicans***

**ERG11_D116E**

In the mutant, the substitution is on an active site residue but it is not a vital amino acid as per our prediction.D116 is situated on a helix and have two vital residues TYR118 and HIS120 within 4 Å of it (FIGURE **S3** a). There is a minimal increase in volume and area of the catalytic cleft (1199 Å^3^, 1036 Å^2^) (FIGURE **S3** b). A single polar contact is conserved in the mutant with SER113 but there is an increase in the bond lenghth (from 2.0 Å in wild to 3.0 Å in mutant). Proximity of the Heme is conserved at 12 Å from the site in both proteins. D and E have similar properties as they both are hydrophilic and are negatively charged. However, E is a bit bigger than D. ERG11_ D116E have slightly more energy compared to wild type (negative magnitude of total energy in mutant is less than the wild model) (TABLE S2) owing to marginally higher electrostatic and non-bonded energies.

**
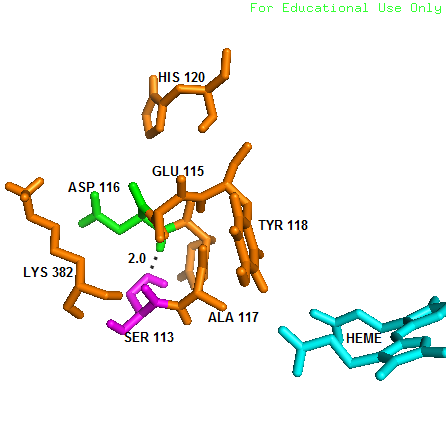

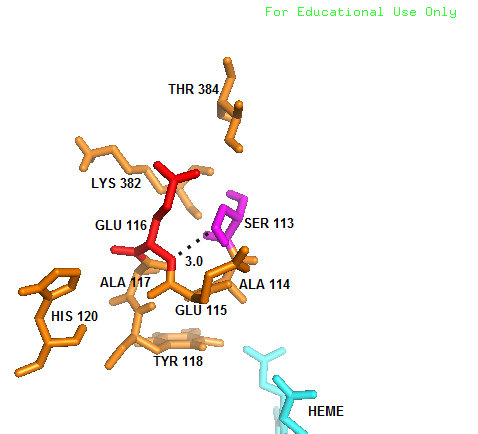
**

**Figure S3 a: Local environment of ASP116 of the wild type ERG11 of *C.albicans* and the GLU116 in the mutant ERG11_D116E**

**
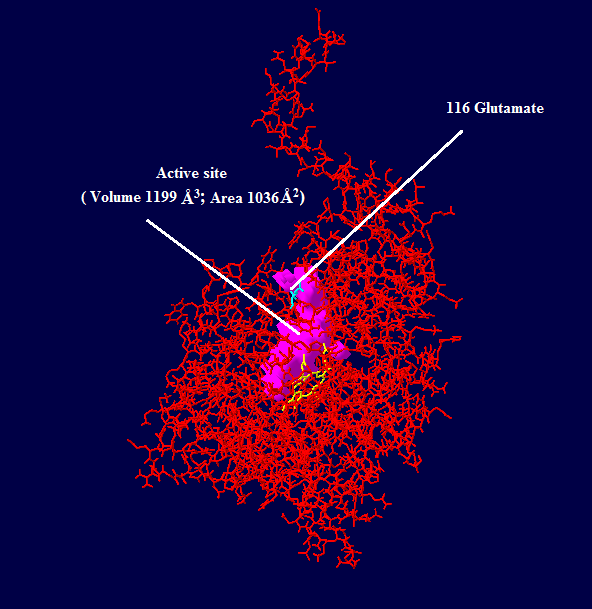
**

**Figure S3 b: Volume and Area of the catalytic pocket of the mutant ERG11_D116E**

**ERG11_Y118A**

Tyrosin 118 is a vital active site residue and situated on a helix. The wild type Y118 has a polar contact (1.9 Å) with PHE380, a vital residue for interaction (FIGURE **S3 c**). The wild residue also has four more polar contacts with four other residues including Leu121 a vital residue. Within 4 Å proximity Tyr118 have three more vital residues His120 , Tyr132 and Phe233.The Heme is within 4 Å of Tyr118.In the mutant the striking alterations are loss of polar contact with vital Phe380, significant change in Heme proximity to 8 Å . Bond length of two polar contacts with Glu115 and Ala114 has been increased significantly. Substitution at this site (Y118A) leads to highest increase in the volume and area of the active site cleft to 1306Å^3^ and 1080 Å^2^ compared to the wild type (FIGURE **S3 d**). The big hydrophobic and aromatic ring of Y118 makes many interactions (FIGURE 2a). The residue must be important for correct binding of the substrate. The mutation changes this big residue into the smaller Alanine. It has a very small side chain and not likely to make the same stabilizing interactions.The energetic stability is also reduced in the mutant (TABLE S2).

**
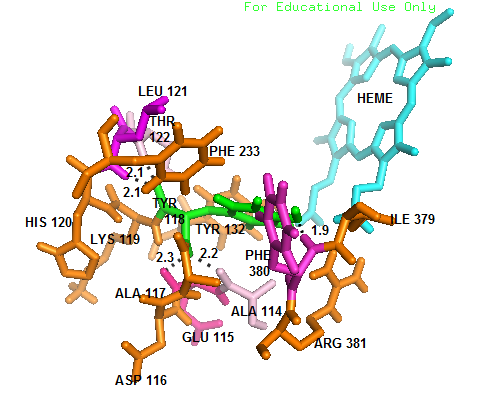

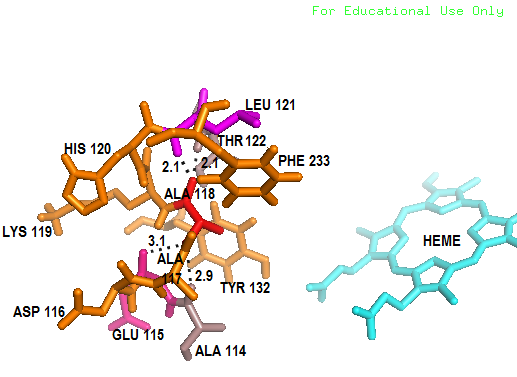
**

**Figure SF3 c: Local environment of TYR118 of the wild type ERG11 of *C.albicans* and the ALA118 in the mutant ERG11_Y118A**

**
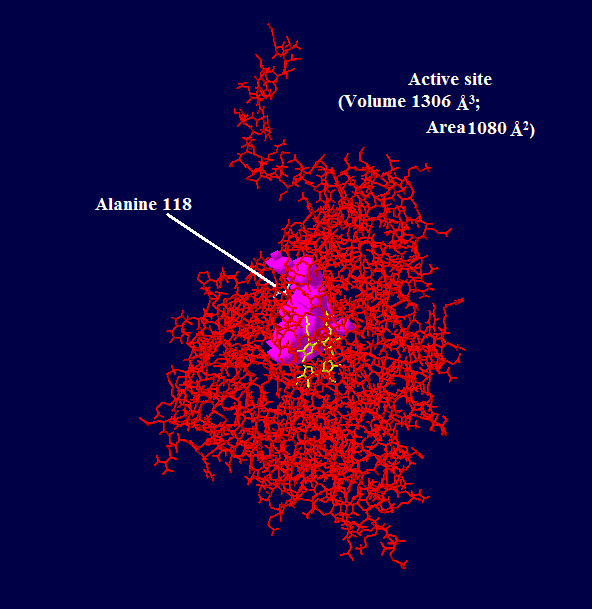
**

**Figure SF3 d: Volume and Area of the catalytic pocket of the mutant ERG11_Y118A**

**ERG11_P230L**

P230 is situated on a helix and is a vital residue for interaction that occurs at the anterior part of the catalytic pocket. Proline is the only residue that interacts with its own backbone and is therefore very rigid and can make angles that other residues cannot make. There is no local changes observed in the mutant (FIGURE **S3 e**) however stability of the protein is decreased as evident from the total energy (-24829.008Kj/mole) (TABLE S2). Also the Proline side chain can make hydrophobic interactions. Due to the mutation, the sharp angle in the backbone may be lost and this will affect the local structure. Active site volume and area is found to be decreased in the mutant (1137 Å^3^, 1000 Å^2^) (FIGURE **S3 f**).

**
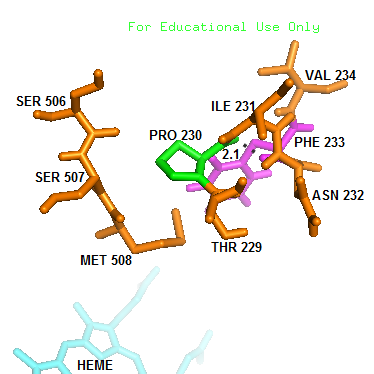

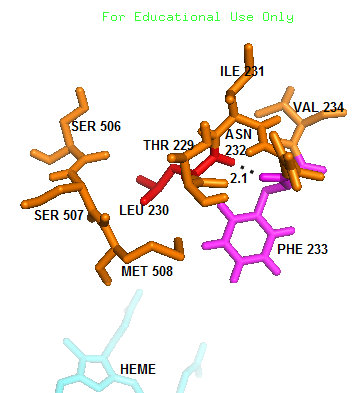
**

**Figure S3 e: Local environment of PRO230 of the wild type ERG11 of *C.albicans* and the LEU230 in the mutant ERG11_P230L**

**
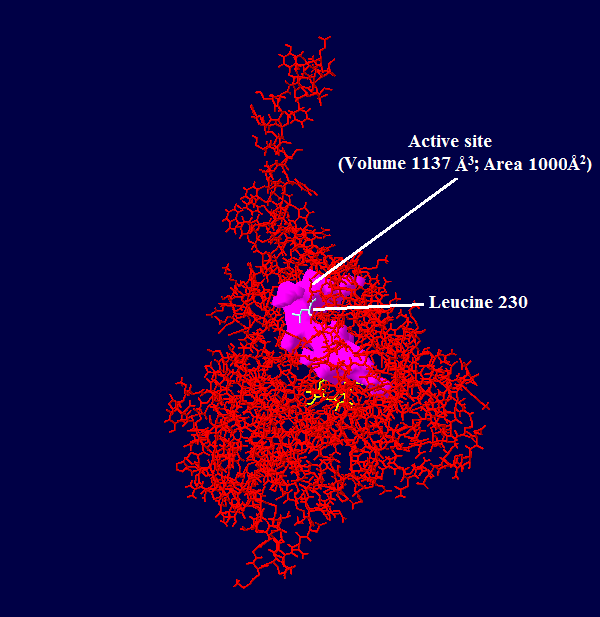
**

**Figure S3 f: Volume and Area of the catalytic pocket of the mutant ERG11_P230L**

**ERG11_K342R**

K342 is on a loop, far away from the active site. There are no functional residues in its proximity. The Heme group is very far from this site (more than 20 Å away) (FIGURE **S3 g**) .The wild type parameters of the active site geometry (volume of furrow 1190 Å^3^  and area 1013 Å^2^) was conserved in the single mutant (FIGURE **S3 h**).The wild and mutant residues at this distant site are similar and both are positively charged. However, the mutation have made the protein more stable (-25108.711 Kj/mole) compared to the wild type with significant decrease in total energy (TABLE S2).

**
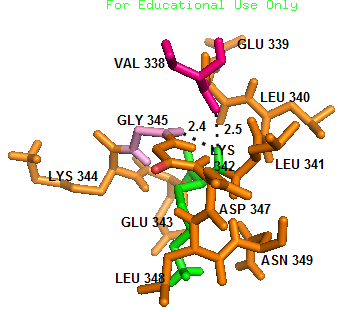

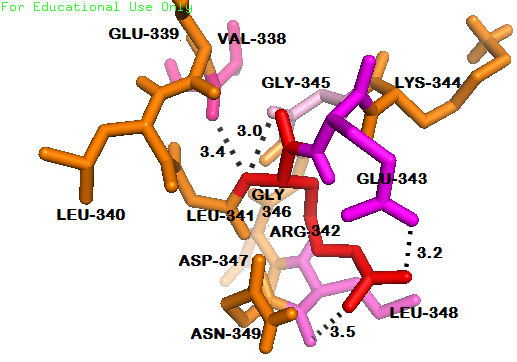
**

**Figure S3 g: Local environment of LYS342 of the wild type ERG11 of *C.albicans* and ARG342 of mutant ERG11_K342R. Note HEME is not visible.**

**
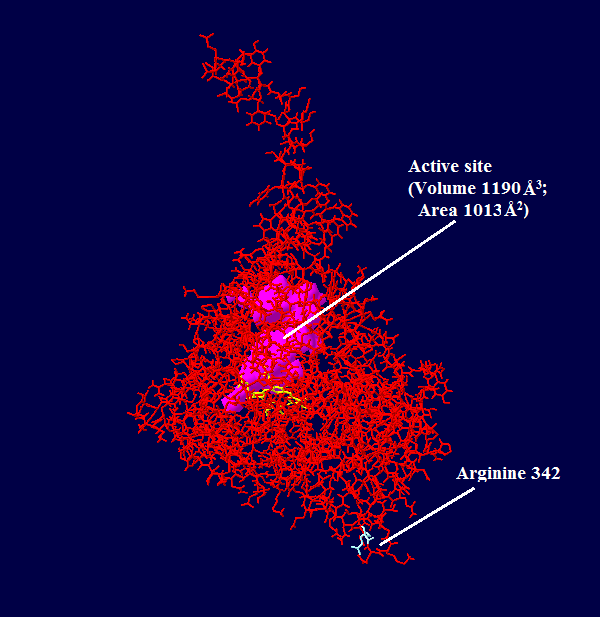
**

**Figure S3 h: Volume and Area of the catalytic pocket of the mutant ERG11_K342R**

**ERG_F380S**

F380 is situated on a beta strand and is a big, aromatic and hydrophobic residue. It’s a vital amino acid for interaction and it’s side chain is lining the pocket and would make several hydrophobic interactions with the Lanosterol. The Heme is within 5 Å of this site. Mutation of this residue into the smaller and less hydrophobic Serine will cause loss of probable hydrophobic interactions and might affect Lanosterol binding. Moreover, F380 have a polar contact (1.9 Å) with another vital residue Tyr118. Two more vital residues Tyr64 and Phe233 are within 4A of the wild F380.The significant alteration of the polar contact (bond length increased to 2.8 Å) as well as the missing vital residues (Tyr64 and Phe233) from the vicinity (4Å) in the mutant will have impact on enzyme function (FIGURE **S3 i**) . Its substitution causes significant increase in the active site volume and area (1294 Å^3^, 1048 Å^2^) (FIGURE **SF3 j**).Energy values indicates that the mutant protein is less stable (-24737.490 Kilo jule/mole) than the wild type(TABLE S2).

**
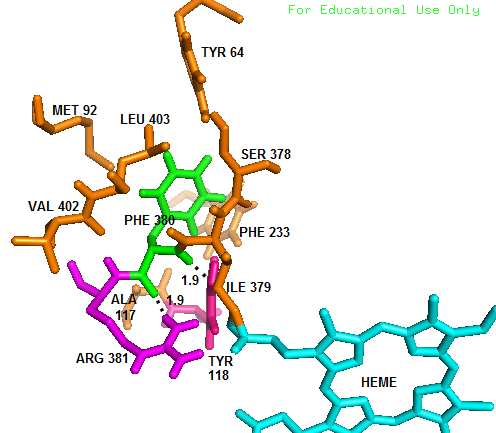

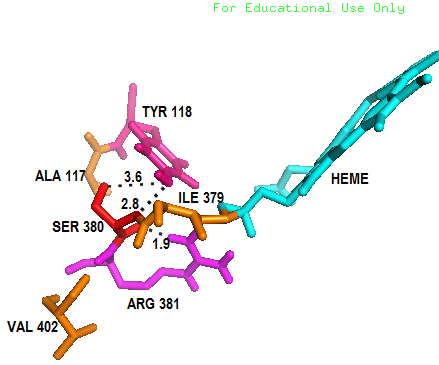
**

**Figure S3 i: Local environment of PHE380 of the wild type ERG11 of *C.albicans* and SER380 of mutant ERG_F380S.**

**
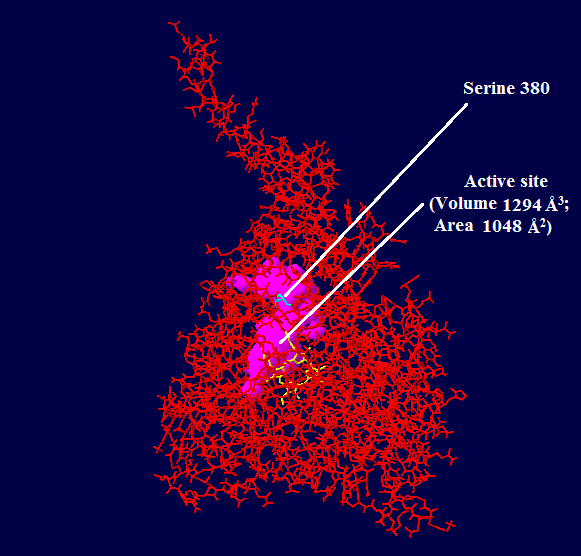
**

**Figure S3 j: Volume and Area of the catalytic pocket of the mutant ERG11_K342R**

**Multiple mutants on ERG 11 of *Candida* *albicans***

**ERG11_ A114S_Y257H**

A114 and Y257 are situated on helix and are neither vital residues and nor in the active site. A114 makes a polar interaction with the vital residue Tyr118 which is maintained in the wild type. Heme is within 5A from this site even in the mutant.

At the mutation site Y257 there are no vital residue in the vicinity of 4A and no polar contacts are made with important functional residues (FIGURE **S3 k,l**) .The volume and area of the catalytic furrow remains conserved (1190 Å^3^ , 1013 Å^2^) as in the wild type (FIGURE **S3 m**).The double mutant do not alter any significant characteristic of the protein, however there is small decrease in stability (-24705.412 KJ/mole) (TABLE S2).

**
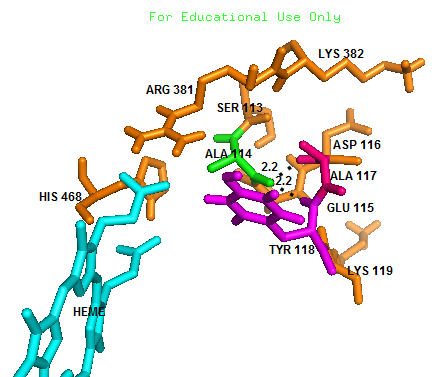

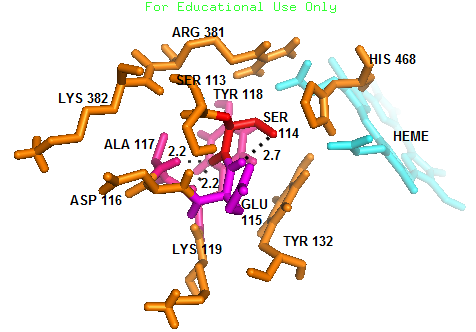
**

**Figure S3 k: Local environment of ALA114 of the wild type ERG11 of *C.albicans* and SER114 of mutant ERG11_ A114S_Y257H**

**
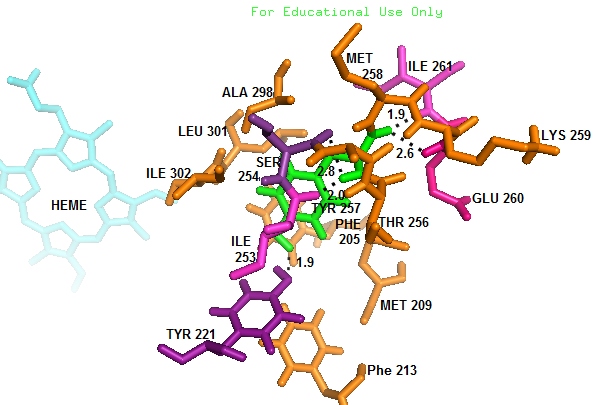

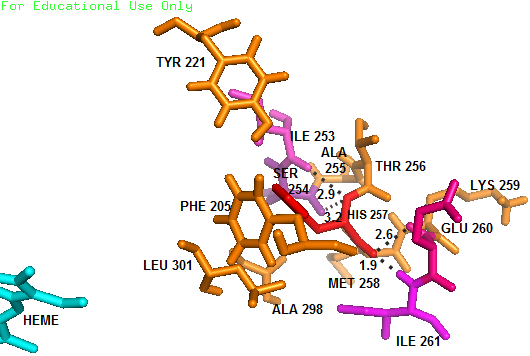
**

**Figure S3 l: Local environment of TYR257 of the wild type ERG11 of *C.albicans* and HIS257 of mutant ERG11_ A114S_Y257H**

**
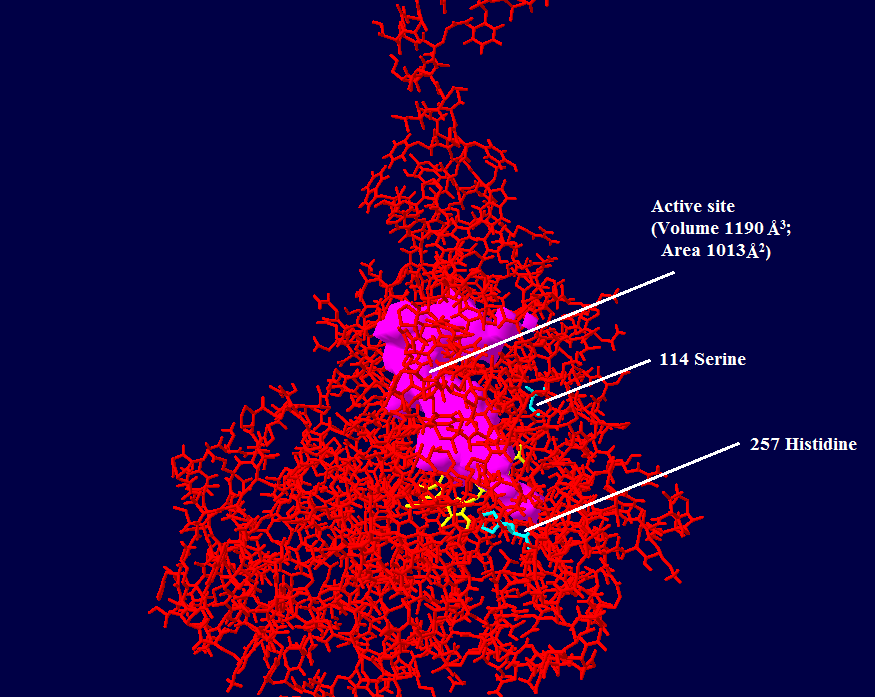
**

**Figure S3 m: Volume and Area of the catalytic pocket of the mutant ERG11_ A114S_Y257H**

**ERG11_D116E_K128T_Y132H_G465S**

In this quadruplet mutant a vital residue Y132 and an active site residue D116 are substituted. Substitution of the active site residue D116 did not altered local environment significantly, but may have contributed to energetic stability (FIGURE **S3 n**). K128 is a non active site residue on a loop which is at far position from the Heme (20 Å) and the active site (FIGURE **S3 o**). The local changes due to a substitution do not influence any of the vital residues or proximity of Heme. Y132 is situated on a loop near Heme. Substitution of the vital active site residue Y132 seems to alter the local environment in a way that influences active site and vital residues as well as the prosthetic group Heme. In the wild protein Y132 have a polar contact (1.6 Å) with O2D atom of the Heme (FIGURE **S3 p**).The vital active residue Y118 and active site residues Lys119, are within vicinity of 4 Å. The mutation at Y132 leads to several local changes out of which most significant is the abolished polar contact with Heme prosthetic group. G465 is situated on a loop. Substitution of G465 do not alter local environment significantly as Glycine and serine are the among the smaller amino acids and their substitution may not have altered the geometry significantly (FIGURE **S3 q**). The active site character is altered with an increase in volume and area (1216 Å^3^,1042 Å^2^) (FIGURE **S3 r**). According to energy scores, this mutant remains most unstable (-23972.287Kj/mole) (TABLE S2).

**
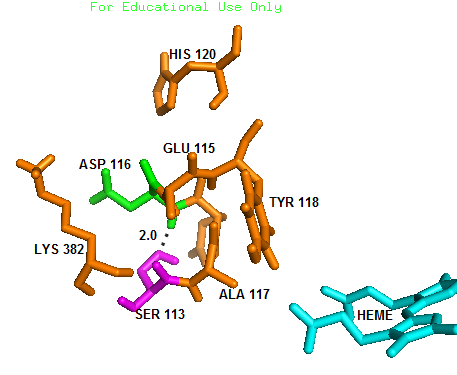

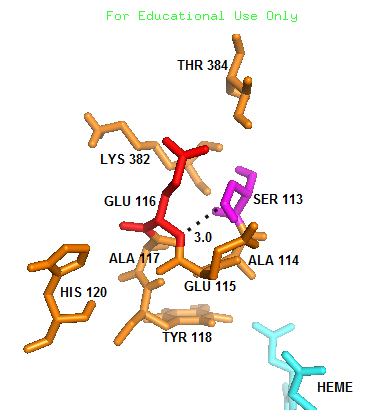
**

**Figure S3 n: Local environment of ASP116 of the wild type ERG11 of *C.albicans* and GLU116 of mutant ERG11_D116E_K128T_Y132H_G465S**

**
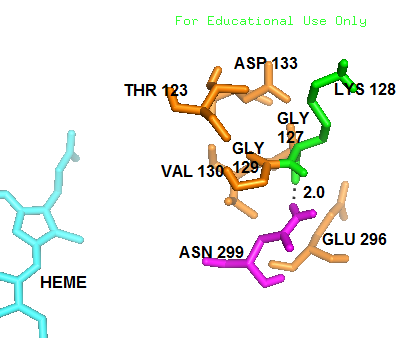

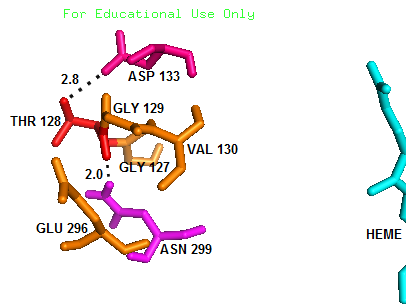
**

**Figure S3 o: Local environment of LYS128 of the wild type ERG11 of *C.albicans* and THR128 of mutant ERG11_D116E_K128T_Y132H_G465S**

**
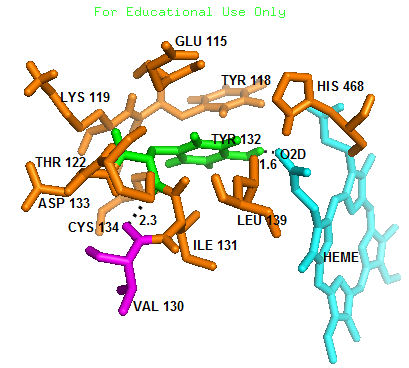

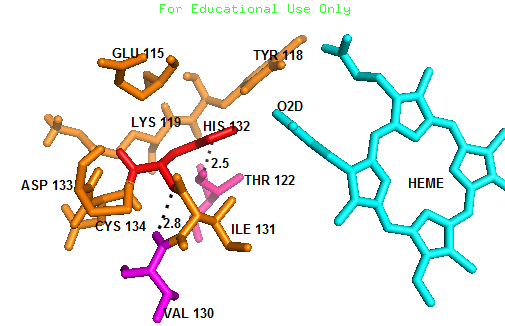
**

**Figure S3 p: Local environment of TYR132 of the wild type ERG11 of *C.albicans* and HIS132 of mutant ERG11_D116E_K128T_Y132H_G465S**

**
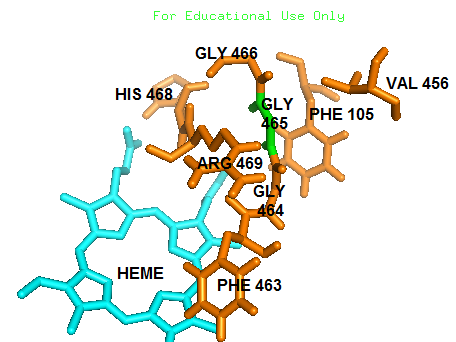

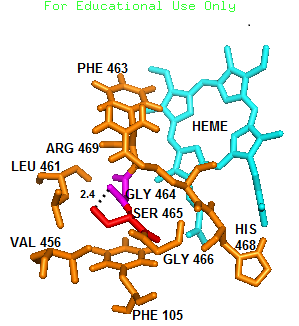
**

**Figure S3 q: Local environment of GLY465 of the wild type ERG11 of *C.albicans* and SER465 of mutant ERG11_D116E_K128T_Y132H_G465S**

**
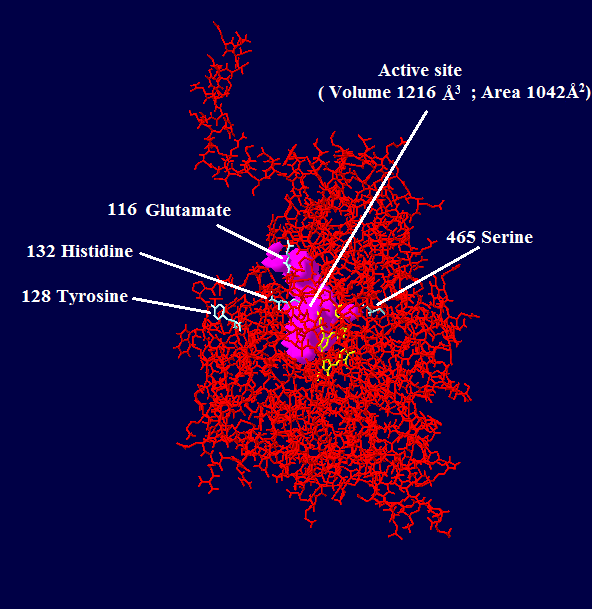
**

**Figure S3 r: Volume and Area of the catalytic pocket of the mutant ERG11_D116E_K128T_Y132H_G465S**

**ERG11_Y132H_G450E**

In this double mutant substitution of the vital Y132 substantially alters local environment by abolishing polar contact with Heme. The side chain of Y132 points towards both the active site and the Heme group (FIGURE **S3 n**). Histidine is known to do be used as an electron storage system in several other proteins and its presence in the active site may alter the electrical environment. Substitution of G450 altered the amino acids in the vicinity; also length of the single polar bond is increased in the mutant. Glycine is the smallest residue and doesn’t have a side chain. (FIGURE **S3 s**) The residue can often be found at places where no other residue would fit. The E side chain is comparatively bigger and carries a negative charge. In this double mutant active site geometry altered by changing its volume and area (1202 Å^3^, 1021 Å^2^) (FIGURE **S3 t**).The protein is unstable compared to the wild protein with total energy -24604.473 KJ/mole (TABLE S2).

**
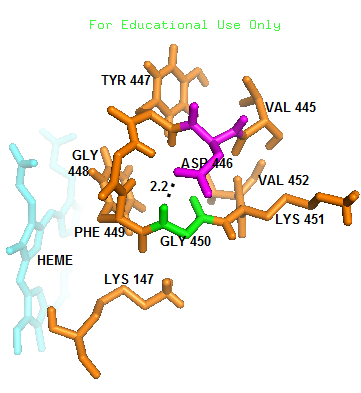

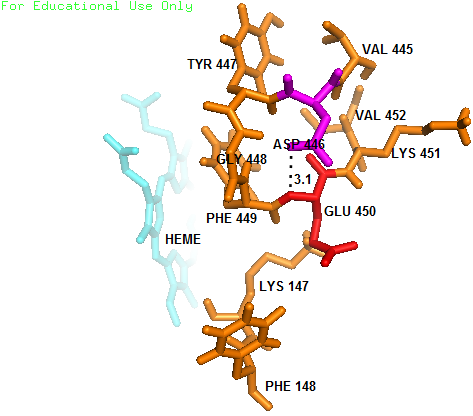
**

**Figure S3 s: Local environment of GLY450 in wild type ERG11 of *C.albicans* and GLU450 in ERG11_Y132H_G450E**

**
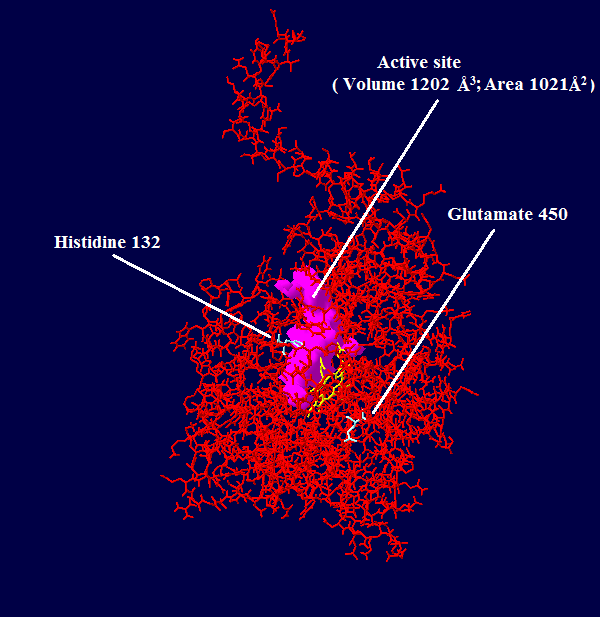
**

**Figure S3 t: Volume and Area of the catalytic pocket of the mutant ERG11_Y132H_G450E**

**Comparative account of the dynamic behavior of the ERG11 mutants with reference to the wild ERG11.**

The Mean Square Displacement 〈*R*^2^〉 of wild type ERG11 is plotted on top and serves as the reference value. Subsequent plots are different mutants of ERG11.

**
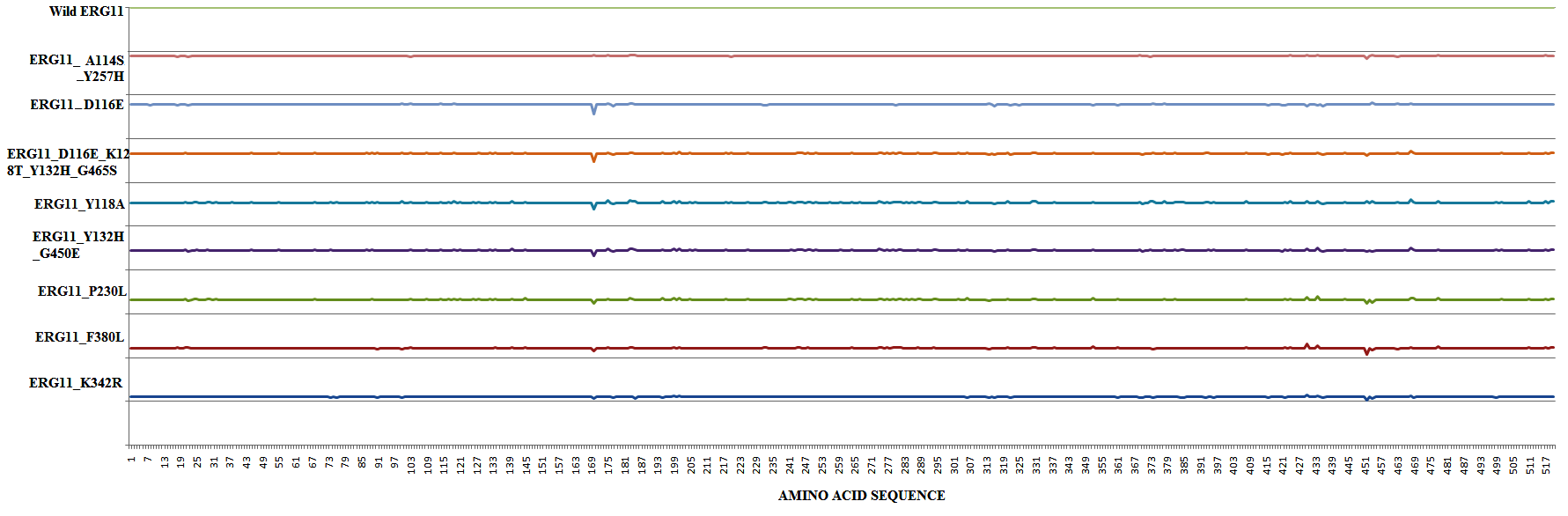
**

**Figure S4: Mean Square Displacement 〈*R*^2^〉 of wild type ERG11 and various mutants with reference to the ERG11 wild type. Minimal fluctuations were recorded for the mutants ERG11_ A114S_Y257H and ERG11_K342R**

**Mutated Amino acid residues within 4Å of the Heme prosthetic group of ERG11 in *C.albicans* .**

The table below describes the mutants with substitution on amino acids in close proximity to the ERG11 prosthetic group. The table was prepared after extensive literature review.Among 24 amino acid residue around the Heme only six amino acids have been found to be substituted in clinical isolates of *Candida albicans.Substitutions at* Y118 and T315 are experimentally found to cause resistance but not isolated from clinical samples**.**

**Table S 3 : Amino acids within 4Å of the Heme prosthetic group of ERG11 in *C.albicans* reported to be mutated .**

| **Sl.no** | **Mutations** | **References** | **Comment** | **Functional Nature Substituted Residues** | |
| --- | --- | --- | --- | --- | --- |
|  |  |  |  | **Active site residues** | **Vital residues** |
| **1** | F105L | Loffler et al., 1997 | Described in Azole-resistant strains as a single mutation or in combination with amino acid changes involved in Azole resistance | None | None |
| **2** | Y118A / Y118F/ Y118T | Chen et al., 2007; Lamb et al., 1997 | The mutation has been clearly associated with resistance by experimental methods but has not yet been detected in clinical isolates. | Y118 | Y118 |
| **3** | K143R/ K143E | Favre et al., 1999; Goldman et al., 2004 | Have been recovered exclusively from Azole-resistant strains and their involvement in Azole resistance has been confirmed using in vitro experiments (heterologous gene expression, affinity between Azoles and CA-CYP51 and significantly increase FLZ MIC). | None | None |
| **4** | I304N | Sanglard and Bille, 2002 | Detected in clinical isolates. Described in Azole resistant isolates. | None | None |
| **5** | G307S | Chau et al., 2004; Goldman et al., 2004;  Perea et al., 2001 | Mutation only described in Azole-resistant isolates. | None | None |
| **6** | T315A | (Chen et al., 2007; Lamb et al., 1997). | The mutation has been clearly associated with resistance by experimental methods but has not yet been detected in clinical isolates. | None | None |
| **7** | G464S | Chau et al., 2004; Franz et al., 1998;  Li et al., 2004; Loffler et al., 1997;  Marichal et al., 1999; Perea et al., 2001;  Sanglard et al., 1998 | Have been recovered exclusively from Azole-resistant strains and their involvement in Azole resistance has been confirmed using in vitro experiments (heterologous gene expression, affinity between Azoles and CA-CYP51). Associated with resistance. Responsible for a 64-fold increase in FLZ MIC. | None | None |
| **8** | I471T | Kakeya et al., 2000; Xu et al., 2008  Sanglard and Bille, 2002 | Have been recovered exclusively from Azole-resistant strains and their involvement in Azole resistance has been confirmed using in vitro experiments (heterologous gene expression, affinity between Azoles and CA-CYP51). | None | None |

**
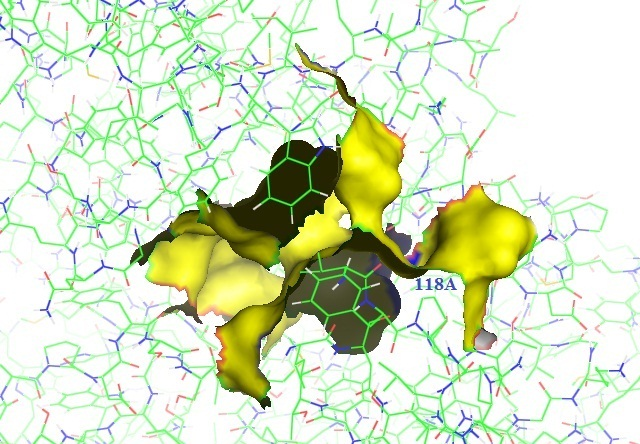

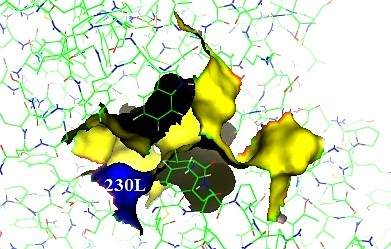
**

**
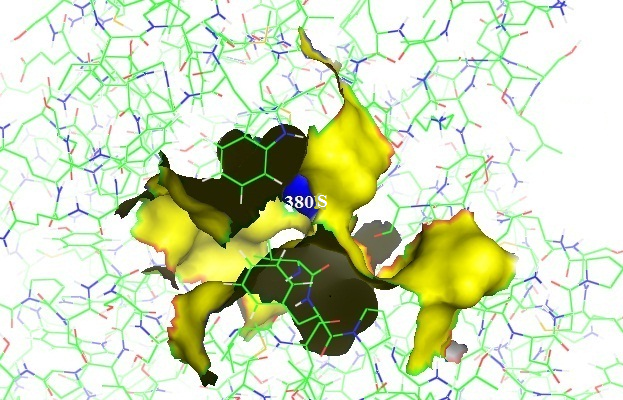
**

**Figure S 5: Surface generated on the vital residues after substitutions in mutants ERG11_Y118A , ERG11_P230L and ERG_F380S.**
